# Super‐Enhancer Driven LIF/LIFR‐STAT3‐SOX2 Regulatory Feedback Loop Promotes Cancer Stemness in Head and Neck Squamous Cell Carcinoma

**DOI:** 10.1002/advs.202404476

**Published:** 2024-08-29

**Authors:** Jin Li, Yuhan Wang, Ziyu Wang, Yuxiang Wei, Pengfei Diao, Yaping Wu, Dongmiao Wang, Hongbing Jiang, Yanling Wang, Jie Cheng

**Affiliations:** ^1^ Department of Oral and Maxillofacial Surgery The Affiliated Stomatological Hospital of Nanjing Medical University Jiangsu 210029 China; ^2^ Jiangsu Key Laboratory of Oral Disease Nanjing Medical University Jiangsu 210029 China; ^3^ Jiangsu Province Engineering Research Center of Stomatological Translational Medicine Nanjing Medical University Jiangsu 210029 China

**Keywords:** cancer stem cells, head and neck squamous cell carcinoma, leukemia inhibitory factor, stemness, super‐enhancer

## Abstract

Super‐enhancers (SEs) have been recognized as key epigenetic regulators underlying cancer stemness and malignant traits by aberrant transcriptional control and promising therapeutic targets against human cancers. However, the SE landscape and their roles during head and neck squamous cell carcinoma (HNSCC) development especially in cancer stem cells (CSCs) maintenance remain underexplored yet. Here, we identify leukemia inhibitory factor (LIF)‐SE as a representative oncogenic SE to activate LIF transcription in HNSCC. LIF secreted from cancer cells and cancer‐associated fibroblasts promotes cancer stemness by driving SOX2 transcription in an autocrine/paracrine manner, respectively. Mechanistically, enhancer elements E1, 2, 4 within LIF‐SE recruit SOX2/SMAD3/BRD4/EP300 to facilitate LIF transcription; LIF activates downstream LIFR‐STAT3 signaling to drive SOX2 transcription, thus forming a previously unknown regulatory feedback loop (LIF‐SE‐LIF/LIFR‐STAT3‐SOX2) to maintain LIF overexpression and CSCs stemness. Clinically, increased LIF abundance in clinical samples correlate with malignant clinicopathological features and patient prognosis; higher LIF concentrations in presurgical plasma dramatically diminish following cancer eradication. Therapeutically, pharmacological targeting LIF‐SE‐LIF/LIFR‐STAT3 significantly impairs tumor growth and reduces CSC subpopulations in xenograft and PDX models. Our findings reveal a hitherto uncharacterized LIF‐SE‐mediated auto‐regulatory loop in regulating HNSCC stemness and highlight LIF as a novel noninvasive biomarker and potential therapeutic target for HNSCC.

## Graphical abstract

SOX2/SMAD3 preferentially binds to LIF‐SE which recruits BRD4/EP300 to activate LIF transcription; LIF produced by cancer cells and CAFs activates LIFR/gp130‐STAT3 signaling which in turn promotes SOX2 transcription by STAT3 binding in its promoter region, thus forming a previously unknown feedback loop to augment LIF and facilitate stemness in HNSCC. Pharmacological targeting LIF‐SE‐LIF/LIFR‐STAT3 axis represents a novel, viable strategy for HNSCC CSC eradication.

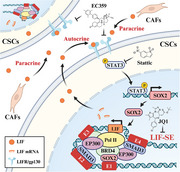

## 1 Introduction

Head and neck squamous cell carcinoma (HNSCC), the sixth most common cancer worldwide, significantly contributes to the global cancer burden and poses great medical and socioeconomic challenges.^[^ ^1^ ^]^ Locoregional relapse, cervical lymph node metastasis, and therapeutic resistance are largely responsible for treatment failure and cancer‐related mortality in HNSCC.^[^ ^2^ ^]^ Mounting evidence has demonstrated that a small, unique subpopulation of cancer cells at the apex of the cell hierarchy, termed cancer stem cells (CSCs) or tumor‐initiating cells (TICs), harbors potent self‐renewal and tumorigenic capabilities and fuels tumor overgrowth, metastatic spread, immune escape and therapeutic failure across various human cancers including HNSCC.^[^ ^3^, ^4^ ^]^ Various innovative therapeutic strategies targeting CSCs alone or in combination with other therapeutic approaches have been developed and shown remarkable anti‐cancer effects and translational potentials in multiple preclinical models.^[^ ^5^, ^6^ ^]^ However, the intrinsic molecular mechanisms and regulatory circuits underlying CSC maintenance and stemness regulation in HNSCC remain incompletely known, thus hampering further diagnostic, prognostic, and therapeutic optimizations.

Transcriptional dysregulation has been increasingly recognized as a universal hallmark of human malignancy.^[^ ^7^, ^8^ ^]^ Enhancers are the noncoding cis‐regulatory elements critically involved in the transcriptional activation of lineage‐specific oncogenes to maintain malignant traits in cancer.^[^ ^9^, ^10^ ^]^ Noticeably, super‐enhancer (SE) has been coined to define a subset of enhancers stitched together which is markedly enriched in higher levels of histone modifications (H3K27ac, H3K4me1) and dense occupations of Mediator complex, EP300, Bromodomain‐Containing Protein 4 (BRD4) as well as key transcription factors (TFs).^[^ ^10^, ^11^, ^12^ ^]^ Several lines of evidence have revealed that SEs preferentially and robustly drive aberrant transcription of essential oncogenes to maintain cell identity and malignant features across multiple cancers.^[^ ^13^, ^14^ ^]^ For example, SE‐driven aberrant transcriptions of SOX2, TP63, AP‐1/FOSL1, and KLF4 maintained CSCs self‐renewal and tumor‐seeding potentials and conferred CSC‐like properties to differentiated non‐CSCs in diverse cancers.^[^ ^15^, ^16^, ^17^, ^18^, ^19^, ^20^, ^21^ ^]^ Moreover, chemical or genetic disruption of SE and SE‐associated transcriptional partners selectively impaired tumor growth and metastasis in vitro and preclinical animal models. We and others have reported that therapeutic perturbations of SEs by various BRD4/EP300/CDK7 inhibitors effectively eliminated CSC subpopulations and strongly impaired invasive growth, recurrence, and metastasis across multiple human cancers including HNSCC.^[^ ^13^, ^14^, ^22^ ^]^ These preclinical findings highlight the essential functions of SE in CSC stemness maintenance and underscore the high values of SE‐targeting strategies against these devastating entities.^[^ ^16^, ^17^, ^23^ ^]^ Nevertheless, detailed SE‐driven transcriptional regulatory circuitries governing CSC properties in HNSCC deserve further in‐depth explorations.

Leukemia inhibitory factor (LIF), a member of IL‐6 superfamily, serves as a pleiotropic cytokine that regulates cell renewal, proliferation, and differentiation in both embryonic and adult tissues. It binds with its receptors LIFR/gp130 and then activates JAKs‐STAT3 signaling to execute its biological roles.^[^ ^24^ ^]^ Accumulating evidence has indicated that LIF functions as a putative pro‐oncogene involved in cell proliferation, dissemination, and CSCs properties and serves as a novel prognostic biomarker and promising therapeutic target across diverse cancers.^[^ ^25^, ^26^, ^27^, ^28^ ^]^ Moreover, previous studies have offered clues to support the pro‐tumorigenic roles of LIF underlying HNSCC initiation and progression.^[^ ^29^, ^30^ ^]^ However, the precise regulatory mechanisms of LIF underlying HNSCC CSC remain underexplored yet.

Here, we characterized HNSCC enhancer and SE landscape via genome‐wide H3K27ac ChIP‐seq profiling and identified LIF‐SE as a novel oncogenic SE driving LIF transcriptional activation. Our results revealed that LIF promoted CSC stemness via LIF/LIFR‐STAT3‐SOX2 axis, while SOX2 facilitated LIF transcription via binding to LIF‐SE, thus forming a previously unknown regulatory feedback loop. Furthermore, our findings from preclinical models and clinical samples underscored LIF as a potent prognostic biomarker and promising therapeutic target for HNSCC.

## 2 Results

### 2.1 Identification and Characterization of Enhancer Landscape in HNSCC

**Figure 1:**
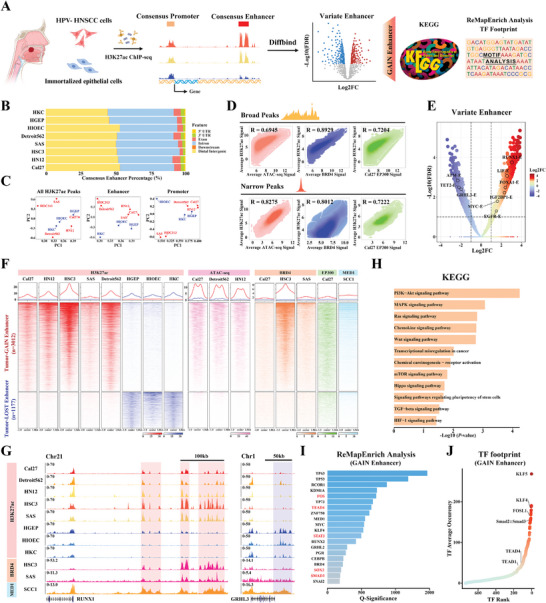
Characterization of enhancer landscape and their functional annotations in HNSCC. A) Schematic description of enhancer profiling in HNSCC through H3K27ac ChIP‐seq and subsequent functional analyses; B) Genome‐wide annotations of enhancer locations in 5 HNSCC cell lines and 3 non‐tumor cell lines; C) PCA analyses to classify HNSCC cells and non‐tumor cells using signals of total H3K27ac peaks, consensus enhancers, and consensus promoters; D) Correlation among average signals of H3K27ac, ATAC‐seq, BRD4, and EP300 binding at these consensus enhancers regions. Signals were extracted by board/narrow peaks and subjected to Spearman's correlations; E) Consensus enhancers were re‐centered and differential analysis of consensus enhancers by using the DiffBind algorithm was performed. Enhancers were named as their assigned genes; F) Heatmap plot displaying the signal enrichments of H3K27ac, ATAC‐seq, BRD4, EP300, and MED1 in GAIN‐E and LOST‐E regions; G) Three representative GAIN‐E located upstream of the RUNX1 gene and one representative LOST‐E located downstream of the GRHL3 gene were illustrated; H) KEGG analysis of GAIN‐E associated genes; I) GAIN‐Es were queried against the ReMap2022 database of human ChIP‐seq datasets to compute the TF or epigenetic regulator binding enrichment. Representative TFs or epigenetic regulators were highlighted in red; J) TF footprint analyses on GAIN‐Es were employed via the RGT‐HINT pipeline. The average occurrence of TFs across 5 HNSCC cells was ranked. Representative TFs were highlighted.

Given the functional importance of enhancer dysregulation as a key epigenetic hallmark underlying HNSCC, we leveraged multiple H3K27ac ChIP‐seq datasets (5 HNSCC cell lines and three non‐tumor epithelial cell lines) to delineate genome‐wide enhancer profile and characterize their biological roles as schematically outlined in **Figure** **1**A. Active enhancers were defined by H3K27ac peaks ±3 kb away from transcription start sites (TSS). As expected, most of these enhancer elements were located at intron and distal intergenic regions (Figure 1B). Diverse numbers of enhancers were detected among these cells. Cancerous cells harbored much longer enhancer elements compared to non‐tumor cells (Figure S1, Supporting Information). Noticeably, we applied principal component analyses (PCA) to cluster these cell lines based on total H3K27ac signals, enhancers, and promoters, respectively.^[^ ^31^ ^]^ As shown in Figure 1C, these cell lines can be readily separated into cancerous or non‐tumor clusters only by enhancers but not total H3K27ac peaks or promoters, indicative of these H3K27ac‐defined enhancers as distinguishing features between HNSCC and non‐tumor cells. In addition, we pooled these constitute enhancers as consensus enhancers and found that H3K27ac enrichments and ATAC signals were strongly correlated at both broad and narrow peaks at these consensus enhancer locus (Spearman's correlation, broad peaks: R = 0.6945; narrow peaks: R = 0.8275). Likewise, these H3K27ac peaks highly correlated with signals of two canonical enhancer‐associated markers BRD4 and EP300 (Spearman's correlation, board peaks: R = 0.8929, 0.7204; narrow peaks: R = 0.8012, 0.7222, Figure 1D). To identify tumor‐specific enhancer alterations, we utilized DiffBind algorithm for variate enhancer calling (|logFC|≥1, FDR<0.05) and found 4089 variant enhancer loci (VEL), including 3012 GAIN Enhancers (GAIN‐E) and 1177 LOST Enhancers (LOST‐E) (Figure 1E). As anticipated, these GAIN‐E displayed increased chromatin accessibilities as well as increased BRD4, EP300 and MED1 binding signals (Figure 1F). Representative GAIN‐E (RUNX1 loci) and LOST‐E (GRHL3 loci) were shown with characteristic and enriched H3K27ac/BRD4/MED1 occupancies (Figure 1G). Next, we assigned VEL to their closest genes via the HOMER algorithm and characterized their biological roles via Kyoto Encyclopedia of Genes and Genomes (KEGG) analyses. These GAIN Enhancer‐associated genes were significantly enriched in multiple HNSCC‐associated pathways including PI3K‐AKT, MAPK signaling, and transcriptional misregulation in cancer, and others (Figure 1H). Moreover, we performed ReMapEnrich analyses to predict binding enrichments of TF in these GAIN‐E loci and found several TFs like AP‐1 (FOS), SMAD3 and TEAD4 potentially binding these loci (Figure 1I). In parallel, similar findings were recapitulated via digital TF footprint analysis at these GAIN loci (Figure 1J). Indeed, these TFs have been well established as oncogenic factors driving HNSCC development.^[^ ^17^, ^32^, ^33^ ^]^ Taken together, our data mining and bioinformatics analyses highlighted aberrant enhancer profiles and their associated transcriptional dysregulations underlying HNSCC initiation and progression.

### 2.2 Identification and Characterization of SE Landscape in HNSCC

**Figure 2:**
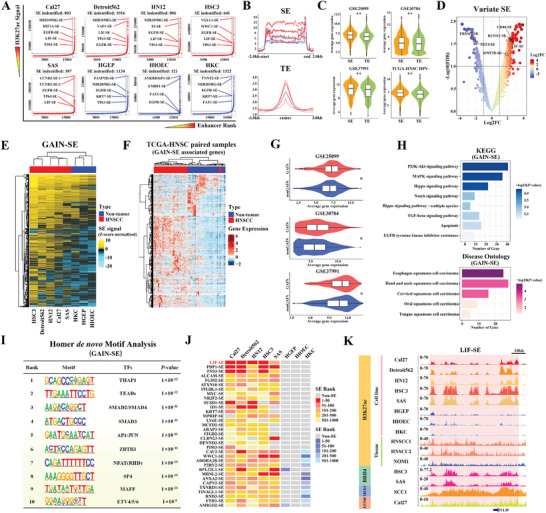
Characterization of SE landscape and identification of LIF‐SE in HNSCC. A. Enhancer clusters in each cell line were identified by the ROSE algorithm using a 12.5 kb window and ranked by summarized H3K27ac signals. Enhancer clusters above or below the inflection point of the curve were defined as SE or typical enhancer (TE), respectively; B) Metagene plots illustrated the H3K27ac enrichment at TE and SE regions; C) The average expression of these TE‐ and SE‐associated genes in 4 HNSCC datasets was compared; D) Differential analysis of consensus SEs was performed via the limma algorithm. SEs were named as their assigned genes; E) The normalized H3K27ac signals of GAIN‐SEs were clearly segregated between HNSCC cell lines and these non‐tumor cell lines using the unsupervised consensus clustering method; F) The expression profile of GAIN‐SE associated genes can segregate HNSCC samples from their paired non‐tumor counterparts using the unsupervised consensus clustering method using TCGA‐HNSC dataset (*n* = 34 pairs); G) The average expression of GAIN‐SE‐ and nonGAIN‐SE‐associated genes in 3 HNSCC datasets were compared; H) Pathway enrichment analyses of GAIN‐SE associated genes via KEGG and DO algorithms; I) *De novo* motif discovery at GAIN‐SE regions via HOMER algorithm; J) Heatmap plot displaying the rank of indicated GAIN‐SEs among HNSCC and non‐tumor cell lines. LIF‐SE had the highest rank in HNSCC cells but was absent in non‐tumor cells; K) Genomic tracks plot displaying the signals of H3K27ac (8 cell lines, 2 primary HNSCC tissue, and 1 human normal oral mucosa (NOM)), BRD4, EP300, and MED1 binding at the LIF‐SE region. Wilcoxon rank‐sum test. *p < 0.05, **p < 0.01.

SEs have been demonstrated to drive malignant transformation and cancer progression largely by their indispensable roles involved in transcription.^[^ ^10^, ^13^ ^]^ However, SE repertoire in HNSCC and their roles remained underexplored yet. To address this, we utilized the ROSE algorithm which called and stitched H3K27ac enriched enhancers in proximity (a 12.5 kb window as default parameter) based on those H3K27ac ChIP‐seq datasets from cell lines to category enhancers into SE or typical enhancers (TE) (**Figure** **2**A,B).^[^ ^11^ ^]^ The numbers of SE differed remarkably across these cells (Figure S2, Supporting Information). Subsequently, these SE were mapped with their‐associated genes (SE‐associated genes) via combined annotation algorithms. As expected, the average transcriptional levels of these SE‐associated genes were significantly higher than TE‐associated genes across four independent HSNCC cohorts (Wilcoxon rank‐sum test, Figure 2C). As shown in Figure 2D and Figure S3A (Supporting Information), 1149 GAIN‐SE and 627 LOST‐SE were found as variant super‐enhancers loci (VSEL) in HNSCC cells. Representative GAIN‐SE and LOST‐SE in HNSCC cells were shown with characteristic H3K27ac, BRD4, MED1 and EP300 binding enrichments (Figure S3B, Supporting Information). In agreement with previous notion that SEs were usually found in chromosome regions with mutations and/or amplifications,^[^ ^34^, ^35^ ^]^ both amplified GAIN‐SE or deleted LOST‐SE regions were more frequently detected in copy number variation (CNV) hot‐spot regions including chr3, 7, 8, 11, 13 in HNSCC samples (Figure S4A,B, Supporting Information).^[^ ^36^ ^]^ As shown in Figure S4C (Supporting Information), GAIN‐SE^Amp^‐associated genes displayed the highest mRNA abundance in HNSCC as compared to other gene groups (Wilcoxon rank‐sum test).

Furthermore, both PCA and hierarchical clustering readily separated these cells based on SE signals (Figure 2E; Figure S3C, Supporting Information), suggesting the existence of HNSCC‐specific SE landscape. Complementarily, transcriptional profiles of these GAIN‐SE‐associated genes effectively distinguished tumor samples from their paired adjacent non‐tumor samples in TCGA‐HNSC cohort (Figure 2F). The expression patterns of GAIN/nonGAIN‐SE‐associated genes well corresponded to their SE status in HNSCC samples (Wilcoxon rank‐sum test, Figure 2G). Gene ontology (GO) analyses indicated that these GAIN‐SE‐associated genes were significantly enriched in epithelial proliferation/migration, stem cell proliferation, DNA‐binding as well as transcription‐associated events (Figure S5, Supporting Information). KEGG and Disease ontology analyses revealed significant enrichments of these genes in PI3K‐AKT, MAPK and Hippo pathways and multiple squamous cell carcinomas including HNSCC (Figure 2H). Moreover, motif analyses revealed that multiple TFs like TEADs, AP1 and SMADs were enriched in those SE regions, strongly implying their potential regulatory functions driving transcription of these SE‐associated genes (Figure 2I; Figure S6, Supporting Information)

As shown in Figure 2J, a prominent SE region (chr22:30581493‐30659814) near LIF (hereafter named as LIF‐SE) stood as a prominent and functionally untapped SE in HNSCC as its presence and highest ranks across all HNSCC cell lines examined and the absence in non‐tumor cells. To substantiate the presence of LIF‐SE in HNSCC clinical samples, we performed H3K27ac ChIP‐seq in freshly collected HNSCC (HNSCC1 and HNSCC2) and normal oral mucosa (NOM) samples and identified similar LIF‐SE loci in primary cancer samples only (Figure 2K; Figure S7, Supporting Information). Interestingly, this LIF‐SE region has been identified as an oncogenic SE in multiple cancers such as osteosarcoma and esophageal adenocarcinoma.^[^ ^26^, ^37^ ^]^ Collectively, our findings characterized the aberrant SE landscape in HNSCC and underscored LIF‐SE as a representative GAIN‐SE in HNSCC for further experimental explorations.

### 2.3 LIF‐SE Drives LIF Aberrant Overexpression in HNSCC

**Figure 3:**
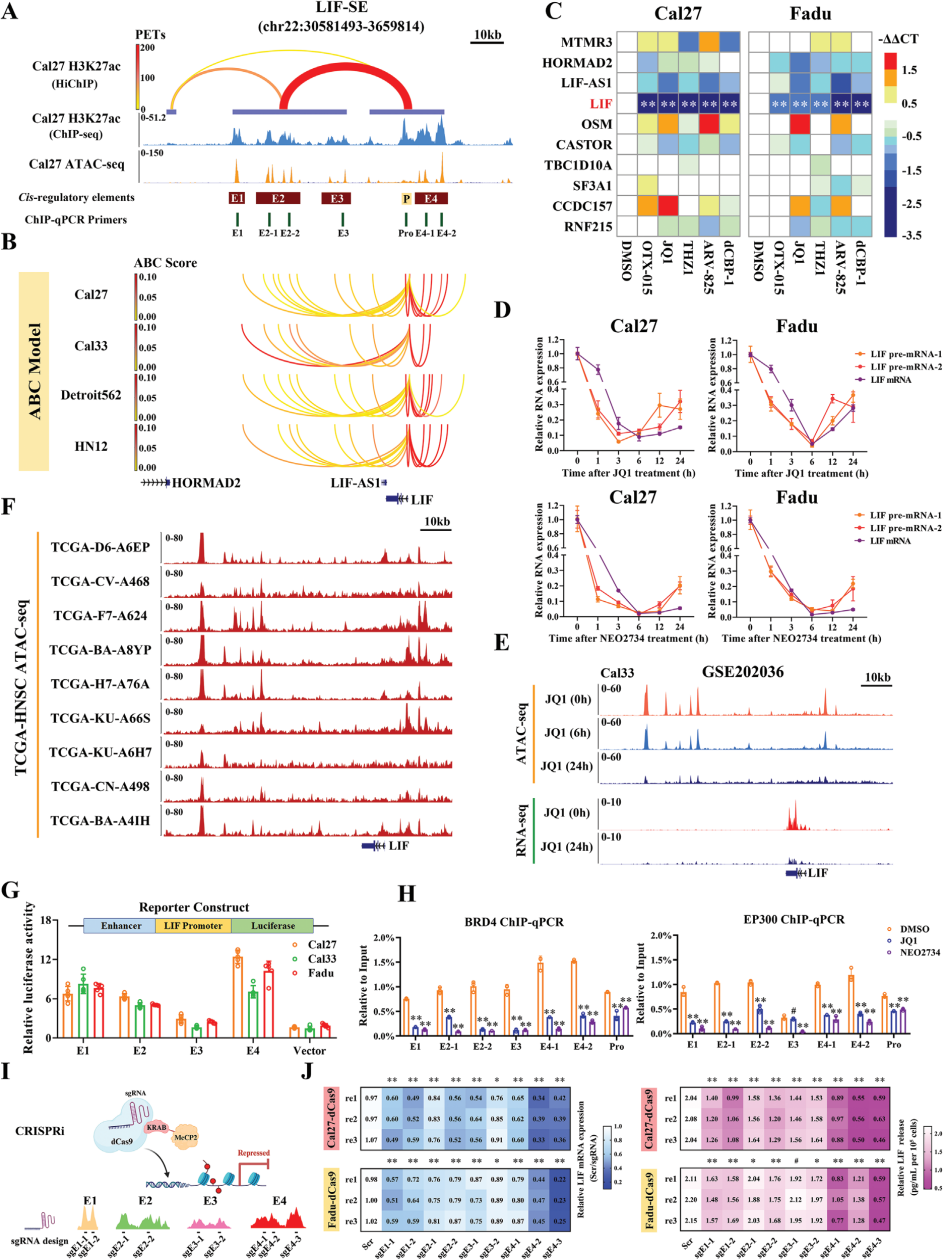
LIF‐SE drives aberrant transcriptional activation of LIF in HNSCC. A. Genomic tracks plots displaying the H3K27ac HiChIP loop, H3K27ac ChIP‐seq peaks, and ATAC‐seq peaks of Cal27 at the LIF‐SE region. LIF‐SE contained five cis‐regulatory elements (referred to as E1, E2, E3, E4 and LIF‐Promoter). Cal27 H3K27ac HiChIP data analysis revealed the connections between enhancer fragments and LIF promoter within LIF‐SE. Our ChIP‐qPCR primers design was illustrated below; B) ABC tracks illustrated that E1‐E4 all have high predicted contact (ABC score) with LIF promoter; C) Heatmap plot displayed the average mRNA changes as measured by qRT‐PCR after indicated epigenetic chemicals treatments in Cal27 or Fadu cells; D) LIF pre‐mRNA and mRNA were measured by qRT‐PCR in Cal27 and Fadu cells treated with JQ1 (1 µm) or NEO2734 (500 n) at indicated time points (0, 1, 3, 6, 12, 24 h); E) ATAC‐seq and RNA‐seq tracks displayed the changes in chromatin accessibility and LIF transcriptional levels after JQ1 treatment in Cal33 cells; F) ATAC‐seq tracks showed the chromatin accessibility of LIF‐SE in HNSCC samples from the TCGA‐HNSC dataset; G) E1‐E4 core enhancer regions were cloned upstream of the luciferase promoter and further used for luciferase reporter assays, respectively; H) The binding changes of BRD4 and EP300 on E1‐E4 and LIF‐promoter after JQ1 exposure (1 µm, 12 h) or NEO2734 (500 nm, 12 h) were measured by ChIP‐qPCR; I) Schematic diagram showing the procedure of E1‐E4 repression by dCas9‐KRAB‐MeCP2 system; J) LIF mRNA levels in Cal27‐dCas9 and Fadu‐dCas9 cells with repressed LIF enhancer activity were assessed by qRT‐PCR (left panel) and LIF titers in supernatants were measured by ELISA (right panel). Student's *t*‐test. *p < 0.05, **p < 0.01.

To further consolidate the LIF‐SE status, we assembled multiple ChIP‐seq datasets and found significant binding enrichments of H3K27ac, H3K4me1, BRD4, MED1, and EP300, but reduced binding of H3K27me3 and H3K36me2 in LIF‐SE across multiple HNSCC cells (Figures 2K and 3A; Figure S8, Supporting Information). Concordantly, increased chromatin accessibilities at this LIF‐SE region were observed in Cal27 cells, indicative of transcriptional activation (**Figure** **3**A). Moreover, Hi‐C dataset analyses indicated high frequencies of internal 3D genome DNA interaction in this loci, while H3K27ac HiChIP dataset analyses suggested substantial enhancer–promoter contacts between LIF promoter and LIF‐SE in HNSCC (Figure 3A; Figures S8 and S9, Supporting Information).

Next, we sought to determine the potential target genes modulated by LIF‐SE by utilizing the activity‐by‐contact (ABC) model.^[^ ^38^ ^]^ Six potential LIF‐SE‐contact genes (LIF, CASTOR, CCDC157, TBC1D10A, SF3A1, and RNF215) were predicted and LIF was the top one with the highest scores among all LIF‐SE enhancer fragments across four HNSCC cell lines (Figure 3B; Figure S10, Supporting Information). Therefore, these results strongly suggest LIF as the primary regulatory target for LIF‐SE in HNSCC. To substantiate the regulatory effects of LIF‐SE on the LIF gene in HNSCC, we initially leveraged multiple chemical compounds to disrupt the global SE function and found that among ten nearby genes of interest (6 potential SE‐contact genes and 4 proximal genes). Genetic manipulation or pharmacological inhibition/degeneration of these SE‐associated co‐activator showed a consistent downregulation of LIF mRNA and protein expression in HNSCC as measured by qRT‐PCR, RNA‐seq, or western blot (Figure 3C, Figure S11). Of note, JQ1 (1 µm) and NEO2734 (500 nm) time‐course treatment (0‐24 h) induced rapid downregulation of both LIF pre‐mRNA and mRNA in HNSCC cells (Figure 3D). Complementarily, JQ1 exposure markedly impaired chromatin accessibility in the LIF‐SE region and LIF mRNA in Cal33 cells measured by ATAC‐seq and RNA‐seq (GSE202036, Figure 3E). Together, these results indicate that LIF‐SE intricately modulates LIF transcription in HNSCC.

Prior reports have documented the diverse roles of individual enhancer elements in SE‐mediated transcriptional activation.^[^ ^15^, ^39^ ^]^ We next set out to pinpoint the detailed roles of these elements involved in LIF transcription. Based on ChIP‐seq and ATAC‐seq datasets, four individual enhancer elements were identified in LIF‐SE (E1‐4, Figures 2, 3). Next, we generated luciferase reporters containing individual E1‐4 gDNA sequences and performed luciferase reporter assays. As illustrated in Figure 3G, E1, 2, 4 consistently induced much higher luciferase activities as compared to E3 or promoter only. In line with this, BRD4 and EP300 occupancies in E1, 2, and 4 were prominently reduced after JQ1 or NEO2734 treatment as assessed by ChIP‐qPCR assay (Figure 3H). As expected, NEO2734 exposure significantly reduced H3K27ac binding in these selected regions while JQ1 failed to exert similar effects (Figure S12, Supporting Information).^[^ ^40^ ^]^ Then, we utilized the improved CRISPR interference (CRISPRi) system (CRISPR‐dCas9‐KRAB‐MeCP2)^[^ ^41^ ^]^ to clarify the contributions of individual enhancers in activating LIF transcription (Figure 3I). Individual repression of E1, 2, 4 by CRISPRi resulted in a remarkable decrease of LIF mRNA expression, accompanied by concomitant reduced protein secretion in culture supernatants (Figure 3J). Taken together, our results indicate that LIF‐SE drives LIF transcription in HNSCC, and SE‐containing E1, 2, 4 might work alone or in combination to orchestrate LIF transcriptional activation.

### 2.4 LIF Promotes CSCs Stemness in HNSCC

Initially, we found the highest percentages of LIF immunohistochemistry staining in HNSCC samples as compared to other cancers using The Human Protein Atlas datasets and aberrant overexpression of LIF mRNA across multiple cancers including HNSCC (Figure S13A–C, Supporting Information). Of note, patients with higher LIF expression had reduced overall survival as compared to those with lower LIF (TCGA‐HNSC: *p* = 0.026, GSE41613: *p* = 0.049, Figure S13D, Supporting Information). These results together with others reinforced that LIF might serve as a putative oncogene and novel prognostic biomarker in HNSCC.

Previous reports have demonstrated that LIF facilitates self‐renewal and stemness in malignant stem cells which are commonly considered as primary culprit for cancer recurrence, metastasis and therapeutic failure.^[^ ^25^, ^28^ ^]^ Inspired by this, we next proceeded to explore the CSCs regulatory functions of LIF and the underlying molecular mechanisms. We calculated the stemness score via ssGSEA and found that LIF mRNA positively corrected with these scores across three independent HNSCC cohorts (Figure S14A, Supporting Information). Additionally, HNSCC samples with top 30% LIF expression harbored differentially expressed genes enriched in CSCs‐related signatures including KRAS signaling and EMT as compared to those with bottom 30% LIF expression (Figure S14B, Supporting Information).

**Figure 4:**
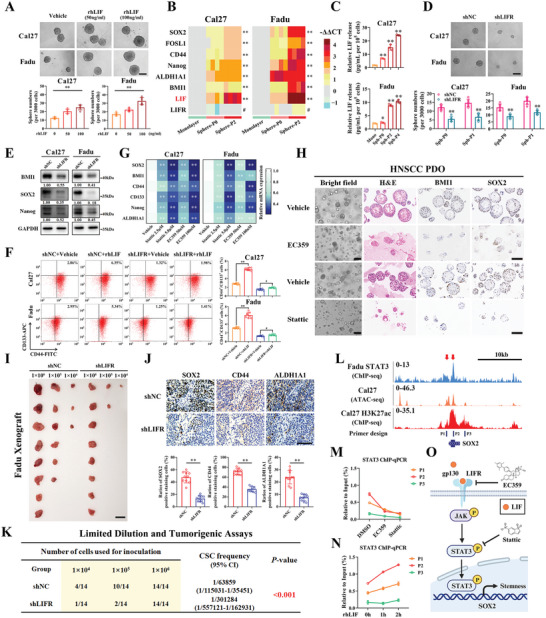
LIF promotes CSCs stemness by STAT3‐mediated transcriptional activation of SOX2 in HNSCC. A) Increased tumorsphere formation was observed in both Cal27 and Fadu cells when exogenous rhLIF was supplemented into the tumorsphere medium. Scale bar: 100 µm; B) The mRNA levels of multiple CSCs markers, LIF, and LIFR in serial passages of Cal27 and Fadu tumorsphere were determined by qRT‐PCR; C) The changes of LIF release in serial passages of Cal27 and Fadu tumorsphere were determined by ELISA assay; D) Impaired tumorsphere formation was observed in stable LIFR knockdown cells. Scale bar: 100 µm; E) The protein abundance of selected CSCs markers was downregulated in LIFR‐deficient cells; F) The CSC (CD44^+^CD133^+^) ratios among Cal27 and Fadu cells under diverse treatments were determined by flow cytometry; G) STAT3 inhibitor Stattic (2.5 µm/5 µm, 48 h) or LIFR inhibitor EC359 (50 nm/100 nm, 48 h) treatment significantly reduced the mRNA expression of selected CSCs markers; H) EC359 and Stattic exposure remarkably impaired PDO growth (measured by brightfield imaging and H&E staining) and reduced SOX2 and BMI1 expression (measured by IHC staining). Scale bar: 100 µm; I) Tumor‐initiating capacities were determined with LIFR‐silencing Fadu cells by limited dilution and tumorigenic assays in vivo. The original image for tumor formation was shown. Scale bar: 1 cm; J) Representative IHC staining images of SOX2, CD44, and ALDH1A1 in xenograft samples derived from Fadu cells with stable LIFR knockdown or controls. Quantification data are shown below. Scale bar: 100 µm; K) CSC frequencies were calculated via ELDA software in pooled data from two batches of in vivo experiments; L) Two prominent STAT3 peaks were identified within the SOX2 promoter using Fadu STAT3 ChIP‐seq dataset (GSE78212). ChIP‐qPCR primers were designed based on STAT3 binding sites; M) The STAT3 binding changes on P1‐P3 after EC359 (100 nm, 12 h) or Stattic exposure (5 µm, 12 h) were measured by ChIP‐qPCR; N) The STAT3 binding changes on P1‐P3 after rhLIF exposure (200 ng mL^−1^, 0, 1, 2 h) were measured by ChIP‐qPCR; O) Schematic illustration of the regulatory model in which SOX2 transcription is regulated via the LIF/LIFR‐STAT3 axis. Student's *t‐*test or One‐way ANOVA test. ^#^ *p* ≥ 0.05, *p < 0.05, **p < 0.01.

We next exploited recombinant human LIF (rhLIF) treatment, siRNA‐mediated knockdown and chemical inhibitors exposure to further reveal LIF's roles in CSCs and pinpoint relevant mechanisms of action. rhLIF exposure remarkably enhanced tumorsphere formation and upregulated stem‐associated markers expression such as SOX2 and BMI1 in Cal27 and Fadu cells (**Figure** **4**A; Figure S15A, Supporting Information). Remarkable increases in intracellular LIF mRNA expression and LIF release in supernatants were observed in 3D tumorsphere culture derived from HNSCC cell lines and patient‐derived cancer cells (PDC) as compared to their corresponding monolayer culture (Figure 4B,C; Figure S15B,C, Supporting Information). Given that LIF binds to gp130 and LIFR to activate the JAK‐STAT pathway,^[^ ^24^ ^]，^ we designed three independent siRNAs targeting LIFR to clarify whether LIF modulated stemness via LIFR. Not unexpectedly, LIFR knockdown significantly impaired tumorsphere formation and downregulated CSCs markers like SOX2, Nanog, CD44 and CD133 in vitro. In addition, cell proliferation, migration invasion capacities were also reduced upon LIFR depletion (Figure 4D,E; Figure S16A–F, Supporting Information). Meanwhile, we utilized two canonical markers, CD44 and CD133, to label CSCs by flow cytometry assays as reported before^[^ ^33^ ^]^ and found that rhLIF addition significantly increased CD44^+^/CD133^+^ proportions while LIFR knockdown reduced the proportions of cells, respectively. However, the re‐addition of rhLIF into stable LIFR‐knockdown cells failed to effectively reverse the effects induced by LIFR depletion as evidenced by minimal changes in CD44^+^/CD133^+^ ratios and tumorsphere‐forming capacities (Figure 4F; Figure S16G,H, Supporting Information). Complementarily, pharmacological inhibition of LIFR by EC359 or STAT3 by Stattic significantly downregulated stemness‐related gene expression, inhibited cell clonal growth and reduced SOX2/BMI1 expression in patient‐derived organoid (PDO) models, as well as compromised tumorsphere formation in vitro (Figure 4G,H; Figure S17, Supporting Information). Next, we employed the limited dilution and tumorigenic assay in vivo to recapitulate these in vitro findings. Various amounts of Fadu cells with stable LIFR knockdown were subcutaneously inoculated into the blanks of nude mice. LIFR silencing markedly impaired tumor formation and decreased CSCs frequency in vivo, accompanied by diminished SOX2, CD44 and ALDH1A1 expression in samples (Figure 4I–K; Figure S18, Supporting Information).

Prior reports have established that SOX2, one of the master transcriptional regulators for stemness, functions downstream of STAT3 signaling to promote CSC maintenance in diverse squamous cancers.^[^ ^42^, ^43^ ^]^ In line with this, we curated the Fadu STAT3 ChIP‐seq dataset and identified two typical STAT3 binding sites at the SOX2 promoter region (termed as P1 and P2, Figure 4L). We designed specific primers flanking each peak, performed ChIP‐qPCR assays and found that rhLIF exposure substantially increased STAT3 binding on both P1 and P2 sites while EC359 treatment decreased its binding (Figure 4M,N). In addition, STAT3 knockdown caused remarkable downregulation of SOX2 (Figure S19A, Supporting Information). Furthermore, rhLIF treatment strongly increased the SOX2 promoter‐dependent luciferase activity in a dose‐dependent manner (Figure S19B, Supporting Information). Of note, LIF and SOX2 mRNA expression were significantly positively correlated in multiple HNSCC cohorts (Figure S19C, Supporting Information). In aggregate, these findings reveal that LIF promotes CSCs stemness via JAK‐STAT3‐mediated SOX2 transcription in HNSCC, thus suggesting that chemical blockade of LIF‐LIFR‐STAT3 might be a promising therapeutic strategy for HNSCC (Figure 4O).

### 2.5 SOX2/SMAD3 Binds to LIF‐SE and Drives LIF Transcription and CSCs Self‐Renewal in HNSCC

**Figure 5:**
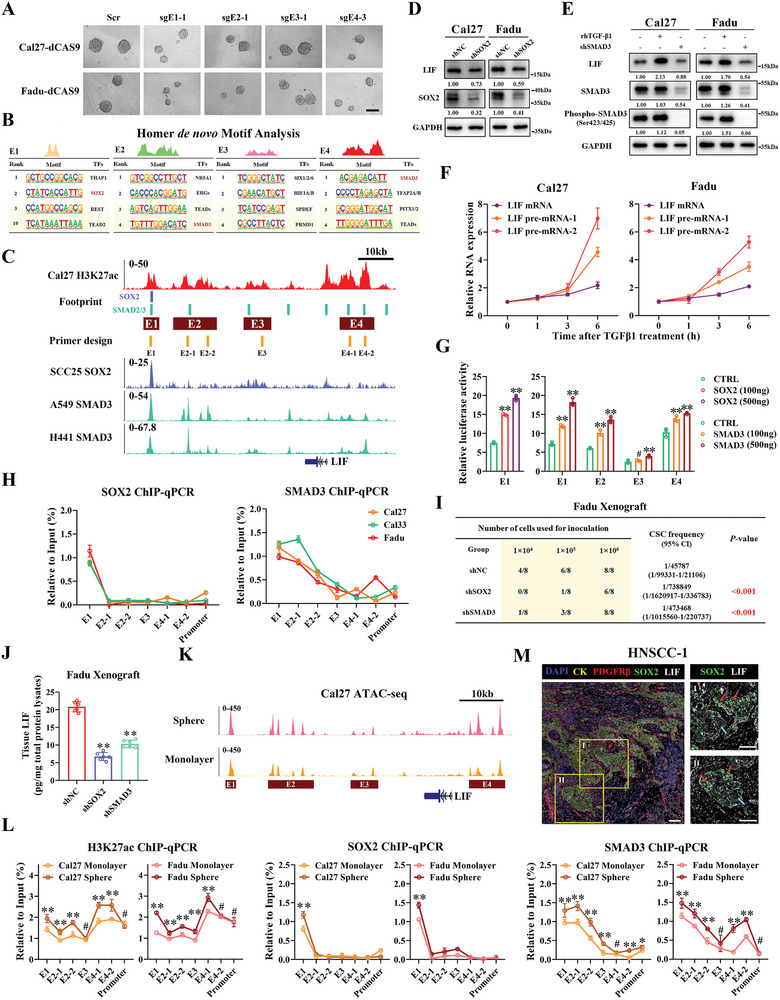
SOX2/SMAD3 facilitates LIF transcription and CSCs stemness via binding with LIF‐SE in HNSCC. A. Representative images of primary tumorsphere formed by indicated enhancer‐repressed tumor cells; B) *De novo* motif analyses of individual constituent enhancers in LIF‐SE by HOMER algorithm; C) The integrated footprint profiles of SOX2 and SMAD3 derived from ATAC‐seq datasets across 4 cell lines were depicted (upper panel). The SOX2 and SMAD3 binding in selected SCC cell lines were displayed (lower panel); D,E) Endogenous LIF protein changes were measured in Cal27 and Fadu cells upon rhTGFβ−1 exposure (20 ng mL^−1^, 48 h) or SOX2/SMAD3 knockdown; F) LIF pre‐mRNA and mRNA were measured by qRT‐PCR in Cal27 and Fadu cells treated with rhTGFβ−1 (20 ng mL^−1^) at indicated time points (0, 1, 3, 6 h); G) Increased luciferase activities of E1 reporter were observed in HEK293T cells transfected with increased dosages of SOX2 plasmid, while increased luciferase activities of E1, 2, 4 reporters were observed after exogenous SMAD3 overexpression; H) The SOX2 and SMAD3 binding at E1‐E4 and LIF promoter in Cal27, Cal33, and Fadu cells were detected by ChIP‐qPCR; I,J) SOX2 or SMAD3 silencing reduced CSC frequencies and downregulated LIF protein expression in Fadu xenografts; K) Chromatin accessibility in monolayer or 3D sphere cultured Cal27 cells was measured by ATAC‐seq, respectively; L) The relative binding of H3K27ac, SOX2, and SMAD3 in Cal27 and Fadu monolayer or tumorsphere cells was assessed by ChIP‐qPCR; M) Representative mIHC images showed the LIF and SOX2 co‐staining within CK^+^ tumor cells in HNSCC samples. Scale bar: 100 µm. Student's *t*‐test. ^#^ *p* ≥ 0.05, *p < 0.05, **p < 0.01.

Having demonstrated the importance of LIF‐SE underlying LIF transcription in promoting HNSCC CSCs stemness, we next sought to unveil the transcriptional factors critically involved in this process. We initially set out to pinpoint which enhancer elements in LIF‐SE region were required for CSCs renewal. As shown in **Figure** **5**A and Figure S20 (Supporting Information), both the numbers and size of tumorspheres formed by cells with E1, 2, and 4 repressions via CRISPRi approach were substantially reduced relative to those control cells. However, E3 repression had minimal effects on tumorsphere formation, in line with its relatively weak effects on LIF transcription. Next, we performed HOMER *de novo* DNA motif analyses on individual enhancers of LIF‐SE. As illustrated in Figure 5B, the SOX2 motif was significantly enriched in E1, while SMAD2/3 and TEADs in E1, 2, and 4 elements. TF footprint analyses via HINT‐ATAC algorithm revealed similar motif enrichment patterns on these enhancers (Figure 5C upper panel). In parallel, we curated SOX2 and SMAD3 ChIP‐seq datasets from multiple malignant epithelial cells and unveiled consistent binding preferences on these elements (Figure 5C lower panel). Meanwhile, SMAD3 mRNA levels significantly correlated with LIF mRNA across several HNSCC transcriptome datasets (Figure S21A, Supporting Information).

To validate these results, we performed shRNA‐based loss‐of‐function assays in vitro by targeting SOX2 and SMAD3. As expected, SOX2 or SMAD3 knockdown significantly downregulated LIF mRNA and protein expression, while simultaneous silencing of both TFs induced more pronounced effects (Figure 5D,E; Figure S21B, Supporting Information). In contrast, rhTGFβ−1 exposure caused rapid elevations of LIF pre‐mRNA and mRNA in both Cal27 and Fadu cells (Figure 5F). Meanwhile, ectopic SOX2 overexpression increased E1 luciferases activity, while SMAD3 overexpression enhanced E1, 2, 4 luciferases activities (Figure 5G). Importantly, our results from ChIP‐qPCR assays indicated that significant enrichment of SOX2 binding was observed at E1, while SMAD3 occupancy was preferentially enriched in E1, 2, 4 elements (Figure 5H). Moreover, our co‐IP and ChIP‐qPCR results indicated that SOX2/SMAD3 interacted with BRD4/EP300 and their depletions impaired BRD4/EP300 binding at LIF‐SE locus, thus suggesting that SOX2 recruited BRD4 and EP300 to facilitate LIF transcription (Figure S21C,D, Supporting Information).

Given the key roles of LIF, SOX2, and SMAD3 involved in CSC maintenance, we found that tumorspheres formed from SOX2‐ or/and SMAD3‐knockdown cells had markedly reduced LIF protein expression and secretion (Figure S21E, Supporting Information). Moreover, CSC percentages and tumor‐initiating capabilities were markedly reduced in cells with SOX2 or SMAD3 stable knockdown as measured by limited dilution and tumorigenic assays. Reduced LIF abundance was detected in xenograft samples derived from SOX2 or SMAD3‐depleted cells as compared to controls (Figure 5I,J; Figure S22, Supporting Information).

To figure out the mechanisms responsible for LIF enrichment in HNSCC CSCs, we cultured Cal27 cells in monolayer or tumorsphere and collected cells for ATAC‐seq and ChIP‐qPCR assays. The chromosome accessibility at LIF‐SE region was more open in Cal27 tumorsphere than those in monolayer (Figure 5K). Significant enrichments of SOX2 binding at E1 and SMAD3 at E1, 2, 4 were detected in tumorsphere relative to monolayer cells. Similar enrichment of H3K27ac binding further supported differential effects of enhancer elements on LIF transcription in CSCs (Figure 5L). Lastly, we conducted multi‐IHC staining to label SOX2 and LIF expression in HNSCC samples and found that nuclear SOX2 and cytoplasmic staining were frequently observed in individual cancerous cells (Figure 5M). Collectively, our results indicate that SOX2 and SMAD3 preferentially bind to LIF‐SE enhancer elements to activate LIF transcription, which in turn promotes CSC maintenance in HNSCC.

### 2.6 Cancer‐Associated Fibroblasts (CAFs)‐Secreted LIF Promotes HNSCC Stemness

**Figure 6:**
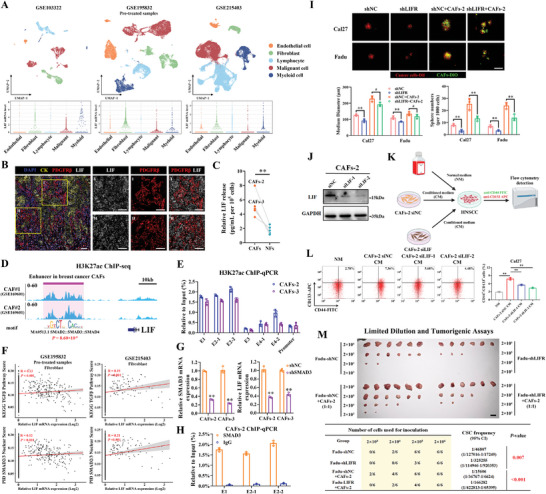
CAFs‐secreted LIF promotes HNSCC stemness by activating LIFR‐STAT3 signaling. A) Uniform manifold approximation and projection (UMAP) visualization and reanalysis of three HNSCC scRNA‐seq datasets (GSE103322, GSE195832 pre‐treated samples and GSE215403) revealed preferential expression of LIF mRNA in malignant epithelial cell and fibroblasts; B) Representative mIHC images showed the positive staining of LIF in PDGFRβ^+^ CAFs in primary HNSCC samples. Scale bar: 100 µm; C) LIF secretion in five paired CAFs and NFs was detected by ELISA; D) Genomic tracks plot displayed the H3K27ac ChIP‐seq peaks in Cal27 and two primary breast cancer CAFs (CAF#1 and CAF#2, GSE169601, no HNSCC CAFs datasets available) at the LIF locus. The ROSE algorithm identified a typical enhancer, but not a SE‐like structure, siting downstream of LIF gene in these CAFs with high SMADs motif enrichments, which corresponded to E1 and E2 regions of LIF‐SE in HNSCC cancer cells; E) The relative binding of H3K27ac in two primary HNSCC CAFs (CAFs‐2 and CAFs‐3) cells was assessed by ChIP‐qPCR; F) The correlations between LIF mRNA and SMAD3‐involved pathway scores (ssGSEA method) in fibroblasts were assessed in two HNSCC scRNA‐seq datasets (GSE195832 Pre‐treated samples and GSE215403). Pearson's correlation; G) The SMAD3 and LIF mRNA levels were measured by qRT‐PCR in SMAD3‐delepted CAFs‐2 cells; H) The binding of SMAD3 and IgG at this CAFs‐specific LIF enhancer was measured by ChIP‐qPCR; I) The indicated DiI‐labeled tumor cells (1000 cells per well, red) were 3D co‐cultured with DiO‐labeled CAFs‐2 (1000 cells per well, green) to form a heterotypic spheroid. Scale bar: 100 µm; J) Endogenous LIF was efficiently silenced by 2 independent siRNAs (siLIF‐1, siLIF‐2) in CAFs‐2 cells. Non‐targeting siRNA was utilized as negative control (siNC); K,L) The percentages of CD44^+^CD133^+^ CSC subpopulations were detected by flow cytometry after Cal27 cells were cultured in normal medium (NM) or conditioned medium (CM) obtained from CAFs‐2 with or without LIF knockdown; M) 10^3^–10^6^ LIFR knockdown Fadu or control cells admixed with or without equal proportions of CAFs‐2 were inoculated in the blank of nude mice. Tumor incidences and growth across four experimental groups were recorded and CSC frequencies in vivo were calculated. Student's *t*‐test. ^#^ *p* ≥ 0.05, **p < 0.01.

The tumor microenvironment (TME) dictates CSC maintenance to orchestrate cancer initiation and progression through intricate crosstalk between CAFs/immune cells and cancer cells.^[^ ^44^ ^]^ Prior studies have provided clues that CAFs secreted LIF to promote malignant phenotypes of oral SCC.^[^ ^30^, ^45^ ^]^ Results retrieved from TIMER2.0 indicated a strong positive correlation between LIF transcripts and CAFs infiltrations in TCGA‐PanCancer datasets (Figure S23, Supporting Information). Next, we exploited three publicly available HNSCC scRNA‐seq datasets (GSE103322, GSE195832 and GSE215403) and determined the preferential expression of LIF among diverse cell subpopulations. As shown in **Figure** **6**A, most LIF mRNA expression were detected in malignant epithelial cells and fibroblasts. mIHC staining of primary HNSCC samples indicated that some PDGFRβ^+^ CAFs had positive, cytoplasmic LIF staining (Figure 6B). Moreover, we cultured five primary CAFs from fresh HNSCC samples and normal fibroblasts (NFs) from paired adjacent non‐tumoral mucosa. ELISA assays revealed more LIF secreted from CAFs in the supernatant as compared with paired NFs (Figure 6C).

To unveil the regulatory mechanisms of LIF in CAFs, we leveraged H3K27ac ChIP‐seq dataset from breast cancer CAFs (GSE169601) but failed to identify similar SE‐like structure at the LIF locus. Instead, a typical enhancer (regions corresponding to E1 and E2 of LIF‐SE in HNSCC cancer cells) was identified the downstream of LIF gene locus (Figure 6D). Subsequently, ChIP‐qPCR experiments in primary CAFs‐2 and CAFs‐3 cells confirmed high enrichments of H3K27ac signals at this enhancer region (Figure 6E). Moreover, motif analysis of this enhancer suggested a high SMAD3 enrichment (Figure 6D). Significant positive correlations between LIF mRNA and SMAD3‐related pathway scores in HNSCC fibroblasts were observed in two scRNA‐seq datasets (Figure 6F). We thus hypothesized that SMAD3 might bind with this LIF enhancer to drive its transcription in CAFs. To experimentally validate this, we conducted siRNA‐mediated SMAD3 knockdown experiment and found that SMAD3 silencing remarkably reduced LIF mRNA level in CAFs. Importantly, ChIP‐qPCR results confirmed SMAD3 binding at this enhancer region, thus suggesting the possibility that SMAD3 was critically involved in LIF transcription in HNSCC CAFs (Figure 6G,H).

Next, we wondered whether CAFs‐secreted LIF functioned to modulate HNSCC stemness in a paracrine manner. To address this, we performed co‐culture assays in vitro via florescence‐labeled Cal27/Fadu cells with or without LIFR knockdown and CAFs‐2 (the CAFs with the highest LIF secretion). Cancer cells co‐cultured with CAFs‐2 significantly enhanced tumorsphere formation in vitro (Figure 6I). Conditioned medium (CM) obtained from control cells significantly increased CD44^+^CD133^+^ fractions in Cal27 cell, while CM from LIF‐silencing CAFs‐2 had compromised capacity to increase CD44^+^CD133^+^ proportions (Figure 6J–L).

Furthermore, limited dilution and in vivo tumorigenic assays revealed that co‐administration of CAFs‐2 robustly promoted tumor formation and increased CSCs frequencies, but failed to induce similar effects in those LIFR‐depleted cancer cells (Figure 6M). Taken together, our findings strongly suggest that LIF either produced by cancer cells or CAFs contributes to HNSCC CSC maintenance in an autocrine/paracrine manner.

### 2.7 Translational Significance of Therapeutically Targeting LIF‐LIFR‐STAT3 Axis in HNSCC

**Figure 7:**
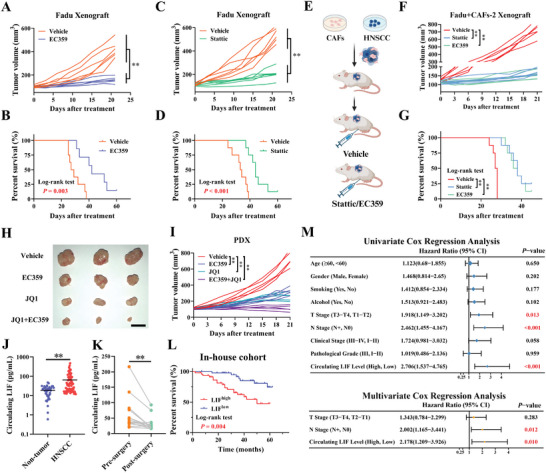
Clinical significance and therapeutic targeting of LIF/LIFR‐STAT3 axis in HNSCC. A‐D) Fadu xenografts were treated with EC359 (5 mg kg^−1^, tiw, i.p.), Stattic (4 mg kg^−1^, qod, i.p.) or vehicle and tumor volume was recorded and compared (A, C). Kaplan‐Meier plots depicted animal survival in both groups after treatments (B, D). Log‐rank test; E) Schematic description of experimental procedures for Fadu admixed with CAFs xenograft model; F,G) Tumor growth curves (F) and Kaplan–Meier plot (G) of Fadu admixed with CAFs xenografts were displayed; H,I) NOD/SCID mice bearing PDX masses were randomly divided into four groups and received with the vehicle, EC359 (5 mg kg^−1^, tiw, i.p.), JQ1 (50 mg kg^−1^, 5 times a week, i.p.) or EC359 plus JQ1 treatment. Tumor mass images after treatments were shown (H). Tumor volume was measured during these treatments (I). Scale bar: 1 cm; J) Circulating LIF levels in non‐tumor individuals (*n* = 40) and HNSCC patients (*n* = 92) were measured and compared; K) Circulating LIF levels in HNSCC patients before or after tumor ablative surgery were measured and compared (*n* = 14). Student's *t*‐test; L) Kaplan‐Meier plots of overall survival in subgroups with high or low circulating LIF levels in our in‐house cohort (Log‐rank test, median level of LIF as cutoff); M) Circulating LIF levels significantly correlated with patients’ survival in our in‐house cohort by both univariate and multivariate Cox regression analyses. **p < 0.01.

To consolidate the translational significance of therapeutically targeting LIF‐LIFR‐STAT3 axis, we developed multiple preclinical models including cell‐derived xenografts and PDX models, and determined therapeutic effects by delivering chemical inhibitors. Intraperitoneal injection of Stattic or EC359 potently diminished tumor growth as evidenced by reduced tumor weights and volume and prolonged animal survival (**Figure** **7**A–D). IHC staining of these xenograft samples indicated that pharmacological inhibition of LIF‐LIFR‐STAT3 axis by Stattic or EC359 significantly reduced SOX2, CD44, and ALDH1A1 expression (Figure S24, Supporting Information). Of note, these treatments were well tolerated without obvious damage to weight gain and vital organs (Figure S25, Supporting Information). Moreover, similar treatments also remarkably retarded tumor growth, and prolonged survival in the CAFs‐Fadu‐admixed xenograft model (Figure 7E–G; Figure S26, Supporting Information). Furthermore, monotherapy with EC359 potently inhibited PDX mass growth and considerably reduced SOX2 and CD44 staining in samples from EC359‐treated mice as compared to those from controls (Figure S27, Supporting Information). To explore whether the synergistic therapeutic effect induced by dual inhibition of SE and LIF/LIFR axis exists, we treated PDX‐bearing mice with intraperitoneal injection of JQ1 and EC359. As expected, combinational treatment resulted in tumor regression and reduced CSC subpopulations compared with either single treatment (Figure 7H,I; Figure S28, Supporting Information). Importantly, both monotherapy and combinational treatments were generally well‐tolerated in animals (data not shown).

Pioneering works have revealed that circulating LIF levels are positively associated with tumor burden and therapeutic outcomes in several cancer contexts.^[^ ^25^, ^27^ ^]^ Given aberrant LIF overexpression in HNSCC, we hypothesized that LIF might be detected in circulating plasma and serve as a novel noninvasive diagnostic or prognostic biomarker for HNSCC. To address this, we collected 92 plasma samples from HNSCC patients before surgery and 40 plasma samples from healthy counterparts and quantitatively measured the circulating LIF via ELISA assays. As shown in Figure 7J,K, circulating LIF protein was readily detected and significantly increased in HNSCC patients and markedly declined after tumor surgical resection. As listed in **Table** **1**, circulating LIF levels were significantly associated with tumor size (*p* < 0.001), cervical node metastasis (*p* < 0.001), and clinical stage (*p* = 0.002) when the median values served as the cutoff. Survival analyses further revealed positive associations between circulating LIF abundance and unfavorable prognosis in our in‐house cohort (Log‐rank test, *p* = 0.004, Figure 7L). Univariate and multivariate Cox regression analyses further established the circulating LIF as an independent prognostic factor after adjustments for multiple clinicopathological parameters (Figure 7M). Collectively, these findings highlight that LIF is a promising prognostic marker and potential therapeutic target in HNSCC.

**Table 1:** Circulating LIF level and the association with clinicopathological parameters in 92 patients with HNSCC.

Parameters		Circulating LIF level		*p*‐values
		Low*	High	
**Gender**	92	46	46	
Male	62	28	34	0.182
Female	30	18	12	
**Age**				
<60	38	20	18	0.672
≥60	54	26	28	
**Smoking**				
No	65	34	31	0.492
Yes	27	12	15	
**Alcohol**				
No	65	34	31	0.492
Yes	27	12	15	
**Tumor size**				
T1‐T2	47	33	14	**<0.001**
T3‐T4	45	13	32	
**Pathological grade**				
I	81	39	42	0.335
II‐III	11	7	4	
**Cervical node metastasis**				
N0	51	32	19	**<0.001**
N+	41	14	27	
**Clinical stage**				
I‐II	39	27	12	**0.002**
III‐IV	53	19	34	

## 3 Discussion

The past decades have witnessed tremendous progress in elucidating the transcriptional deregulation underlying tumorigenesis by identifying various regulatory trans‐ and cis‐elements involved and cis larifying their mechanisms of action.^[^ ^7^, ^8^, ^9^, ^10^ ^]^ In particular, SEs have been demonstrated as novel key elements driving aberrant transcription and maintaining cell identity across multiple human cancers.^[^ ^13^, ^14^, ^16^ ^]^ Here, we comprehensively characterized the aberrant enhancer/SE landscape in HNSCC and identified LIF‐SE as an oncogenic SE promoting LIF transcription, which in turn activated JAK‐STAT3‐SOX2 axis to facilitate CSC maintenance and tumorigenicity in HNSCC. Moreover, SOX2/SMAD3 is preferentially bound to LIF‐SE and activates LIF transcription, thus forming a positive regulatory feedback loop to augment LIF production. Our findings highlight the SEs as key indispensable regulatory components involved in dysregulated transcription and novel prognostic and therapeutic targets in HNSCC.

Enhancers facilitate gene transcription through interactions with promoters and transcriptional apparatus to promote malignant transformation, tumor overgrowth, metastatic spread, and therapeutic resistance.^[^ ^9^ ^]^ Although multiple enhancers have been reported to drive HNSCC progression, genome‐wide enhancers and SE landscapes in HNSCC remained underexplored yet.^[^ ^17^, ^18^, ^20^, ^46^ ^]^ We leveraged H3K27ac ChIP‐seq datasets from multiple cell lines and clinical samples and characterized the HNSCC enhancer/SE landscapes in an unbiased and comprehensive manner. As expected, distinct patterns of enhancer and SE were observed in HNSCC and functional annotations of these enhancer and SE‐associated genes revealed their noticeable enrichments in those well‐known oncogenic pathways and biological categories. Moreover, bioinformatics predictive analyses underscored multiple transcriptional factors like SOX2 and SMAD3 involved in these enhancer‐mediated dysregulated transcriptions. Collectively, our results, together with others reinforced that enhancers/SEs serve as an indispensable layer of complex regulatory networks to facilitate HNSCC progression.

Our SE profiling and calling unveiled LIF‐SE as a prominent but functionally unknown SE in HNSCC. Although similar LIF‐SEs have been identified as pivotal oncogenic SEs in osteosarcoma and esophageal adenocarcinoma, their biological roles and detailed mechanisms of action are still underexplored.^[^ ^26^, ^37^ ^]^ We exploited the ABC model, HiChIP data analysis, and chemical intervention assays to establish that LIF was the primary transcriptional target of LIF‐SE rather than those genes in proximity in HNSCC. Indeed, LIF is a pleiotropic cytokine that regulates stemness in normal stem cell and CSC contexts.^[^ ^24^, ^47^ ^]^ Consistent with these previous results, our results revealed that LIF functioned as a potent factor to maintain CSCs self‐renewal and expansion in HNSCC as evidenced by the following facts: rhLIF exposure promoted tumorsphere formation and CSC markers expression; LIFR knockdown impaired stemness and CSCs proportions; chemical inhibitions of LIFR or STAT3 significantly reduced BMI1 and SOX2 expression in PDO models. Previous reports have established SOX2 as a key lineage‐specific CSCs regulator in HNSCC.^[^ ^42^, ^48^ ^]^ In line with this, our results from ATAC‐seq, ChIP‐seq, and ChIP‐qPCR assays confirmed that STAT3 binding at the SOX2 promoter activated its transcription, which in turn governed HNSCC stemness. Of note, our results also revealed that LIF also critically enhanced HNSCC cell proliferation and invasiveness which deserved further mechanistic explorations. Collectively, our results strengthened that LIF/LIFR activated STAT3 which in turn facilitated SOX2 transcription to promote HNSCC CSC traits.

SE‐containing enhancer elements might not contribute equally to their target transcription.^[^ ^15^, ^39^ ^]^ We pinpointed these LIF‐SE enhancer elements and identified individual E1, 2, 4 as key elements affecting LIF transcription which was also supported by preferential binding enrichments of BRD4 and EP300 in these enhancer elements. However, ATAC‐seq/ChIP‐seq data derived from clinical specimens indicated relatively low levels of chromatin accessibility and H3K27ac enrichments at E4. We reasoned that this discrepancy might be attributed to diverse genetic backgrounds, tumor heterogeneity between clinical samples and cell lines, as well as the limited samples examined. Noticeably, increased chromatin accessibilities and H3K27ac enrichments at E1, 2, 4 regions were found in tumorsphere‐enriched CSC subpopulations compared to monolayer cultured cells, thus reflecting their roles for CSC maintenance. Based on highly complex interactions among SE‐comprising enhancer elements and enhancer–promoter contact, we believe that these E1‐4 might promote LIF transcription in a cooperative manner, although E3 seemed dispensable. Further detailed experiments are warranted to unravel the effects and contributions of individual enhancers on LIF transcription. Collectively, our findings revealed that E1, 2, 4 are predominant enhancers within LIF‐SE to drive LIF transcription which in turn activates STAT3‐SOX2 axis to promote HNSCC CSC maintenance.

SE usually recruits lineage‐specific TFs (SOX2, TP63, FOSL1, and others) and transcriptional co‐activators to cooperatively activate target gene transcription to drive cancer progression.^[^ ^11^, ^12^, ^19^, ^20^, ^21^ ^]^ Our results identified SOX2 and SMAD3 as key TFs to bind LIF‐SE and transcriptionally activate LIF via recruitments of SE‐associated co‐activators like EP300 and BRD4 as transcriptional complexes. However, our findings did not support the direct protein interaction of SOX2 and SMAD3 in HNSCC. Thus, we believe that these two TFs might function independently to activate SE‐mediated LIF transcription in HNSCC. Moreover, we detected strong SMAD3 binding at E1, 2, 4, and promoter and confirmed that SMAD3 promoted LIF transcription largely by its binding in LIF‐SE and to a lesser extent by promoter binding. Prior studies have unveiled LIF as a downstream target of TGFβ signaling and SMAD2/3 binds to LIF promoter and activates its transcription to promote glioblastoma CSCs stemness.^[^ ^28^ ^]^ This different preferential SMAD3 binding at LIF‐SE or promoter between our results from HNSCC and other cancers might be due to diverse genetic background and structure, disease etiology, and others. Collectively, by integrating these mechanistic insights together, we proposed a hitherto uncharacterized SE‐mediated regulatory loop (LIF/LIFR‐STAT3‐SOX2‐LIF‐SE) for HNSCC stemness control. Of course, we cannot rule out that other oncogenic factors (KRAS), TFs (p53), or epigenetic modifiers (UTX) are also involved in LIF transcription in HNSCC as reported among other biological contexts.^[^ ^26^, ^49^, ^50^ ^]^ In aggregate, we conclude that SOX2 and SMAD3 bind to LIF‐SE to activate LIF transcription and thereby facilitate CSC maintenance and tumor development in HNSCC.

Several lines of evidence have indicated that LIF is secreted either by cancer cells and/or CAFs across diverse cancers.^[^ ^30^, ^45^, ^51^, ^52^ ^]^ Indeed, a previous study from Yae Ohata's group has suggested that LIF protein is mainly produced by CAFs in oral SCC,^[^ ^30^ ^]^ while Jean Albrengues reported that LIF protein was abundantly secreted by multiple carcinoma cells including oral SCC.^[^ ^45^ ^]^ Our results reconciled with this discrepancy and revealed that both HNSCC cancer cells (especially those CSCs) and CAFs were able to produce and secrete LIF. Results from multiple HNSCC scRNA‐Seq datasets further supported preferential LIF production in both malignant epithelial cells and fibroblasts. Our results further identified a typical enhancer rather than SE‐like structure downstream of the LIF gene locus in CAFs and indicated that SMAD3 binding in this enhancer promoted LIF transcription in CAFs. Collectively, our findings unveil two independent regulatory models for LIF in HNSCC cancer cells and CAFs to promote LIF transcription, respectively. Noticeably, SMAD3 serves as a key TF involved in LIF transcription in both cancer cells and CAFs via different regulatory models. These LIFs either from cancer cells or CAFs facilitate CSC properties and malignant traits via an autocrine/paracrine manner in HNSCC.

Aberrant overexpression and pro‐tumorigenic functions of LIF were detected across multiple cancers and significantly associated with malignant features and patient survival.^[^ ^25^, ^53^ ^]^ Circulating LIF levels positively correlated with tumor burden and immunotherapeutic outcomes, thus underscoring the translational potential as a noninvasive biomarker.^[^ ^25^, ^29^ ^]^ Consistent with these pioneering studies, our results confirmed higher LIF transcripts in clinical samples and circulating LIF protein in plasma from HNSCC patients. Moreover, LIF titers in plasma were associated with patient survival and served as an independent prognostic factor. Currently, therapeutic targeting of CSCs to improve treatment outcomes has been proposed but remains highly challenging.^[^ ^3^, ^6^ ^]^ Having revealed LIF as a potent CSCs driver, we have found that selective disruption of LIF‐SE or/and LIF/LIFR‐STAT3 potently impaired tumor growth at least in part by reducing CSCs numbers and their tumorigenic activities in multiple preclinical models. Thus, our results together establish that circulating LIF concentration and LIF abundance in clinical samples are promising prognostic biomarkers and LIF/LIFR/STAT3 axis is a druggable target with translational potentials in HNSCC.

Our findings derived from sophisticated bioinformatics data re‐analyses, genetic or pharmacological manipulations in vitro, preclinical cellular/animal models, and clinical samples provide ample experimental support for LIF‐SE‐driven CSC regulatory model in HNSCC. However, our study has several limitations regarding data collection, analyses as well as interpretation. First, limited numbers of clinical samples for enhancer/SE profiling might undermine our abilities to faithfully identify the overall SE landscape in the native HNSCC ecosystem.^[^ ^31^, ^54^, ^55^ ^]^ In addition, interference by other cells from TME (tumor purity) might account for some discrepancies in SE profiling between primary clinical samples and cell lines. Some TME‐derived factors might reshape the SE program in tumor cells.^[^ ^56^, ^57^ ^]^ Second, direct examinations of HNSCC CSC subpopulations might be better and more accurate in uncovering SE candidates essential for their stemness maintenance and control. Single‐cell multiomics profiling might represent an alternative approach to unraveling SE‐associated regulatory functions underlying HNSCC CSC traits. Third, our results provide experimental evidence that LIF derived from cancer cells or CAFs contributes to CSC stemness in HNSCC. However, we cannot confidently determine the source of LIF which plays a dominant role in stemness control, and cannot rule out that LIF from other cell types in tumor microenvironment promotes HNSCC stemness. Many in‐depth mechanistic explorations are warranted to address these limitations.

**Figure 8:**
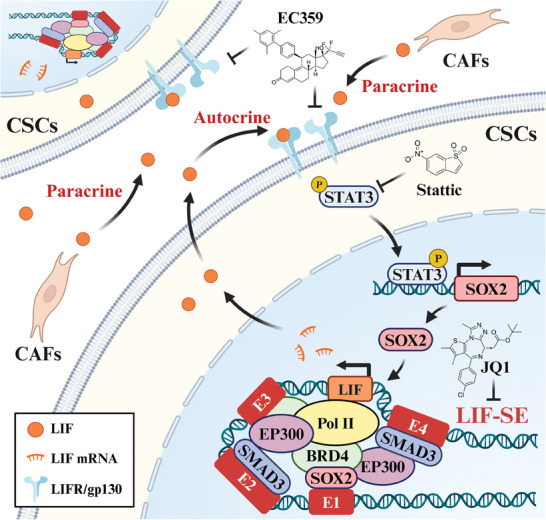
Schematic illustration of LIF‐SE driven LIF/LIFR‐STAT3‐SOX2 regulatory feedback loop to maintain cancer stem cell stemness in HNSCC. SOX2/SMAD3 preferentially binds to LIF‐SE which recruits BRD4/EP300 to activate LIF transcription; LIF produced by cancer cells and CAFs activates LIFR/gp130‐STAT3 signaling which in turn promotes SOX2 transcription by STAT3 binding in its promoter region, thus forming a previously unknown feedback loop to augment LIF and cancer stemness in HNSCC. Pharmacologically targeting LIF‐SE‐LIF/LIFR‐STAT3 axis represents a novel, viable strategy for CSC eradication in HNSCC.

In conclusion, we identified and characterized a novel LIF‐SE‐mediated oncogenic feedback loop comprising LIF/LIFR‐STAT3‐SOX2 to orchestrate CSC maintenance and properties in HNSCC (**Figure** **8**). Our findings further highlight that LIF is a novel biomarker with diagnostic and prognostic significance and targeting this regulatory circuit represents a viable therapeutic strategy for HNSCC.

## 4 Experimental Section

### Data Availability and Collection

All publicly available next‐generation sequencing (NGS) data datasets utilized in this research were retrieved from the NCBI Gene‐Expression Omnibus (GEO) or The Cancer Genome Atlas (TCGA), the detailed information is listed in Table S1 (Supporting Information). Bulk HNSCC transcriptome datasets enrolled were retrieved from TCGA (TCGA‐PanCancer or TCGA‐HNSC) and GEO with accession numbers GSE13601, GSE25099, GSE30784, GSE37991, GSE41613). HNSCC copy number variation data were obtained from TCGA‐HNSC dataset.

### Cell Culture, Transfection and Chemicals

A panel of cell lines, including Cal27, Cal33, Fadu and HEK293T, sourced from China Center for Type Culture Collection (CCTCC, Shanghai, China) was underwent authentication by short tandem repeat (STR) profiling and routinely tested negative for *mycoplasma* contamination. HNSCC cells and HEK293T cells were cultured in DMEM/F12 or DMEM (Thermo, USA), supplemented with 10% FBS (Thermo), under controlled conditions of constant temperature (37 °C) and humidity (5% CO_2_). Cell transfection was performed by Lipofectamine 2000 (Thermo) according to the manufacturer's protocols. Chemical compounds and recombinant cytokines were purchased commercially and their detailed information was listed in Table S2 (Supporting Information).

### Chromatin Immunoprecipitation Sequencing (ChIP‐seq) and ChIP‐qPCR

Fresh samples of HNSCC or non‐tumor oral mucosa tissue (≈2 g) were harvested, washed and ground into powder in liquid nitrogen. Cal27 cells (≈1 × 10^7^) in a logarithmic growth phase with monolayer growth were washed and collected. All samples were fixed with 1% formaldehyde at room temperature for 15 min, followed by 0.125 m glycine treatment for 5 min. The sample was then pelleted, washed, and re‐suspended in ChIP lysis buffer (25 mm Tris‐HCl pH 7.5, 1 mm EDTA, 500 mm NaCl, 1% Triton X‐100, 0.1% sodium deoxycholate, 0.05% SDS, 1 × Proteinase Inhibitor Cocktail). Then, samples were sonicated by Bioruptor Plus sonication device (Shearing condition: 20 cycles on cooling condition, 30 s ON, 30 s OFF), immunoprecipitated with antibody against H3K27ac, BRD4, EP300, SOX2, SMAD3 or Rabbit IgG on ChIP‐Grade Protein G Agarose Beads (#9007, Cell Signaling Technology, USA) and purified by Universal DNA Purification Kit (#DP214, TIANGEN Biotech, China). The ChIP‐Seq library was prepared using NEBNext Ultra II FS DNA Library Prep Kit for Illumina (#E7645L, New England Biolabs, USA) according to the manufacturer's instructions. ChIP‐seq was performed on Illumina Nova‐seq 6000 using a signal‐end model after DNA library ligating with specific adaptors. The raw ChIP‐seq datasets for human samples were uploaded to Genome Sequence Archive (GSA) for Human database (HRA007168). The raw Cal27 BRD4/P300 ChIP‐seq datasets were submitted to GEO database (GSE274995). ChIP‐qPCR was performed on LightCycler 480 System (Roche, Switzerland). The antibodies used for ChIP assay are listed in Table S3 (Supporting Information). ChIP‐qPCR primers used are specified in Table S4 (Supporting Information).

### Assay for Transposase‐Accessible Chromatin with Sequencing (ATAC‐seq)

Monolayer or sphere‐cultured Cal27 cells were trypsinized, counted (≈1 × 10^7^), and lysed in ATAC‐resuspension buffer (0.5% NP‐40, 0.5% Tween‐20, and 0.01% Digitonin). The cell nuclei were then re‐suspended in 50 µL transposase reaction mix (containing 25 µL of 2 × ATAC‐resuspension buffer, 16.5 µL of PBS, 5 µL nuclease‐free ddH_2_O, 0.5 µL of 1% digitonin, 0.5 µL of 10% Tween‐20 and 2.5 µL of Recombinant Tn5 Transposase (Active Motif, USA)) and incubated at 37 °C for 30 min with agitation. After DNA purification, ATAC‐library was prepared and followed by paired‐end sequencing on an Illumina sequencing platform. The raw ATAC‐seq datasets were submitted to the GEO database (GSE274994).

### Real‐Time Quantitative PCR (RT‐qPCR) and RNA Sequencing (RNA‐seq)

RNA‐isolation and were RT‐qPCR performed as previously reported. Briefly, total RNA was extracted using the RNAiso Plus agent (9109, TaKaRa, Japan) according to the manual and followed reversed transcribed into cDNA by PrimeScript RT Master Mix Kit (RR036, TaKaRa). RT‐qPCR was performed on LightCycler 480 System. 2^−ΔΔCT^ method was used for relative quantification. Primers for RT‐qPCR reported in this study are listed in Table S5 (Supporting Information).

For RNA‐seq, a total amount of 3µg RNA per sample was used as input material for the RNA sample preparation. RNA library was prepared and followed by paired‐end sequencing on an Illumina sequencing platform.

### ATAC‐seq/ChIP‐seq/CUT&RUN Data Alignment and Peaks Calling

Raw ATAC‐seq/ChIP‐seq reads were trimmed using Trimmomatic (version 0.39) and aligned using Bowtie2 algorithm (version 2.5.2) to the human reference genome hg19/GRCh37 using the following parameters: ‐t ‐p 36 –local –very‐sensitive‐local –no‐discordant –no‐mixed. BAM format data were sorted and indexed with SAMtools (version 1.18). Peaks calling were utilization the MACS2 (version 2.2.9) algorithm using the following parameters: 1) ChIP‐seq/CUT&RUN: ‐g hs ‐B ‐f BAM –SPMR –keep‐dup all ‐p 1e‐5; 2) ATAC‐seq: ‐g hs ‐B ‐f BAM –shift 100 –extsize 200 –SPMR ‐q 0.01. chrM reads were removed. Pileup signal (.bed files) were converted to binary big‐wig files (.bw file) and Reads Per Kilobase per Million mapped reads (RPKM) normalized by DeepTools (version 3.5.4) using bamCoverage command.

### RNA‐seq Data Analysis

For alignment RNA‐seq data, the pair‐end raw reads of RNA‐seq were trimmed adaptor and aligned to the masked human genome (hg19) by HISAT2 (version 2.2.1). Aligned fragments were assigned to genes by using featureCounts command in subread python package (version 2.0.6).

### Enhancer Peaks Identification and Variate Enhancer Analysis

High confidence H3K27ac peaks (‐log10(*p* value) > 10) of each cell were extracted and annotated by ChIPseeker (R package, version 1.38.0). Peaks located within ±3 kb of TSS region were defined as “Promoter,” whereas others were termed as “Enhancer.” The enhancer/promoter number and average enhancer length were calculated, respectively. Consensus enhancer was obtained by merging enhancer peaks of all cells enrolled using bedtools (version 2.31.0) “merge” command. Enhancer H3K27ac signal normalization and variate enhancer calling was generated by using DiffBind (R package, version 3.12.0) using the following parameters: summits = 350, bUseSummarizeOverlaps = TRUE, method = DBA_DESEQ2, normalize = DBA_NORM_TMM. Enhancer peaks were required to be present in at least 2 of 5 HNSCC cells or one of three non‐tumor cells and indicated cutoff (|Fold| > 1.3, *p* < 0.05) were applied for defined “GAIN/LOST Enhancers (GAIN‐/LOST‐Es).”

### SEs Identification and Variate SE Analysis

SEs were called using the rank ordering of super‐enhancers (ROSE) pipeline by using default parameters (12.5kb‐window) as previously reported.^[^ ^11^ ^]^ For identifying variate SEs across eight cell types, cumulative SE signals were extracted via WiggleTools (Version 1.2), normalized via Z‐Score method, and subsequently undergoing the differential analysis via the limma R package (version 3.58.1). The log2‐normalized signal fold change of SEs more than 0.5 or less than −0.5 were defined as “GAIN‐/LOST‐SE.”

### Assigning Enhancers/SEs to Genes

Enhancers/SEs were assigned to their putative target genes based on the physical interactions (pairs of valid reads (PETs) > 5 and false discovery rate (FDR) < 0.05 between SE and gene promoter in H3K27ac HiChIP chromatin interaction data from Cal27, HN4 and SCC25 cells. In the absence of HiChIP interaction evidence, nearest gene was assigned to the SE using *anntotePeaks* option of ChIPseek R package.

### Analyses of scRNA‐seq Data

Raw gene counts data were downloaded from GEO and quality controls and normalization were carried out using Seurat R package (version 5.1.0). Clusters were identified with the *FindClusters* function at the resolution of 0.1. Then, top expressed genes and known marker genes were used to annotate the main cell types of each cluster.

### Gene Ontology (GO), Kyoto Encyclopedia of Genes and Genomes (KEGG), Disease ontology (DO), Gene Set Enrichment Analysis (GSEA) and ssGSEA

GO, KEGG and DO analyses were performed by using ClusterProfiler R package (version 4.10.0).^[^ ^58^ ^]^

For applying GSEA analyses, differential expression genes (DEG) from the top 30% LIF^high^ and botton LIF^low^ expression patients in TGCA‐HNSC datasets were subjected to ClusterProfiler R package for computing enrichment. Reference gene sets were downloaded from MSigDB (https://www.gsea‐msigdb.org/gsea/msigdb, Hallmark: h.all.v7.2.entrez.gmt; GO: c5.go.v7.2.entrez.gmt; KEGG: c2.cp.kegg.v7.2.entrez.gmt). For calculating stemness scores, ssGSEA method (GSVA, R package, version 1.50.0) was utilized.

### ReMapEnrich Analysis

The ReMap2022 ChIP‐seq data were acquired using the downloadRemapCatalog function available in the ReMapEnrich R package (version 0.99.0).^[^ ^59^ ^]^ Subsequently, the binding of TFs and co‐activators on GAIN‐E regions was analyzed.

### HOMER *De Novo/*Known Motif Analysis

TF motifs were obtained from the JASPAR database (JASPAR 2022 CORE vertebrates). The motif enrichment on indicated genomic regions (GAIN‐Es/GAIN‐SEs) was computed by *findMotifsGenome.pl* module of HOMER (version 4.10) using the default parameters (‐len 8, 10, 12).

### TF Footprint

Footprinting analysis was performed with the RGT‐HINT footprinting pipeline using the following parameters: –atac‐seq –paired‐end (for ATAC‐seq peaks) or ‐histone (for H3K27ac ChIP‐seq peaks).^[^ ^60^ ^]^

### Activity‐by‐Contact (ABC) Model Construction

ABC model (https://github.com/broadinstitute/ABC‐Enhancer‐Gene‐Prediction, version 1.0.0) was utilized to map the enhancer‐gene connections with LIF‐SE in the indicated cell type.^[^ ^38^ ^]^ The top 150 000 strongest narrow peaks from Cal27, Cal33, HN12, or Detirot562 ATAC‐seq were extracted, resized into 500 bp, and then subjected to *makeCandidateRegions.py* for calculating candidate elements. Then, the “neighborhood” genes of each candidate element were retrieved and listed via *run.naighborhoods.py* module. Finally, the chromatin contacts based on power law estimation (options: –score_column powerlaw.Score) were computed by *predicted.py* using a recommended threshold of 0.022. The top 10 target genes of each region with LIF‐SE were selected.

### Plasmid, siRNAs, and shRNA Lentivirus Constructs

Customized siRNAs targeting human LIFR and STAT3 with three independent oligos of each gene used in this research were designed, synthesized, and obtained from GenePharma (China). shSOX2, shSMAD3 and shBRD4 lentivirus constructs have been reported.^[^ ^21^, ^33^ ^]^ siLIFR‐2, the most potency of siRNA sequence, was subcloned into GV248 lentivirus vector (hU6‐MCS‐Ubiquitin‐EGFP‐IRES‐puromycin) for lentivirus production (named as shLIFR). Cal27 and Fadu cells were infected with shLIFR lentivirus in the presence of 5 µg mL^−1^ Polybrene and were further selected with puromycin (5 µg mL^−1^) for 1 week. The efficiency of siRNA‐ or shRNA‐mediated knockdown was verified by qRT‐PCR and western blot, respectively. Detailed sequences of siRNA/shRNA are listed in Table S6 (Supporting Information).

N‐3 × Flag‐SOX2 plasmid was described previously.^[^ ^33^ ^]^ N‐3 × Flag‐SAMD3 was generated by subcloning human SMAD3 cDNA into pcDNA3.1 vector. These vectors were verified by direct sequencing before use.

### CRISPR‐Mediated Enhancer Repression

Cal27 and Fadu cells were transduced with pLV[Exp]‐CBh‐dCas9‐KRAB‐MeCP2:T2A:Hygro (VectorBuilder, China) lentivirus construct for 12 h, allowed to recover for 72 h and followed selected with hygromycin B (200 µg mL^−1^, HY‐B0490, MedChemExpress) for another 7 days. sgRNA were designed to target E1‐E4 on GPP CRISPick webtool (https://portals.broadinstitute.org/gppx/crispick/public) and then synthesized by GenScript (China). A final concertation of 200 nm sgRNA was electroporated into cells via BTX GEMINI System (BTX Technologies, USA). For detecting LIF mRNA expression changes, cells were lysis for RNA isolation after 48 h recovery, while for evaluating LIF release, cells were continuedly cultured for another 72 h. The detailed sequences used are listed in Table S7 (Supporting Information).

### Western blot and Immunoprecipitation (IP)

For the western blot assay, cells were harvested and lysed in cold Western&IP lysis buffer (P0013, Beyotime, China) supplemented with a protease inhibitor cocktail. Protein concentration was determined by the Bradford method and equal amounts of proteins were loaded and separated in 6–12% SDS‐PAGE gels. PVDF membranes (Bio‐Rad) were blocked with 5% (wt/vol) nonfat dry milk in Tris‐buffered saline with Tween‐20, incubated with indicated primary antibodies followed by HRP‐conjugated secondary antibodies and finally detected using on SuperSignal West Pico reagents (34577, Thermo). The relative levels of each protein were quantified with ImageJ software and GAPDH served as loading control. For IP assays, cell lyses were incubated with antibodies and Pierce Protein A/G Plus Agarose (20423, Thermo) overnight at 4 °C. These precipitants were washed five times and eluted with western loading buffer containing 1% SDS for 5 min at 95 °C. Both immunoprecipitated proteins and total lysates were resolved by SDS‐PAGE and immunoblot analyses. Relevant primary antibodies used for western blot and IP assays are listed in Table S3 (Supporting Information).

### Immunofluorescence (IF)

Cells were propagated on glass coverslips in a 24‐well plate for 24 h, washed with PBS and fixed with 4% paraformaldehyde in PBS, permeabilized for 10 min by Immunostaining Permeabilization Buffer with Triton X‐100 (P0096, Beyotime) and blocked in QuickBlock Blocking Buffer (P0220, Beyotime). Cells were further labeled at 4 °C with primary antibodies overnight followed by incubation with fluorescent‐dye‐conjugated secondary antibodies. The nucleus was counterstained with DAPI (C1002, Beyotime). Relevant primary antibodies used are listed in Table S3 (Supporting Information).

### CCK‐8 Assay and Colony Formation

CCK‐8 assay and colony formation assays were performed as we previously reported.^[^ ^33^ ^]^

### Cell Migration and Invasion Assays

Cell migration and invasion assays were performed as we previously reported.^[^ ^33^ ^]^

### Tumorsphere Assay

Tumorsphere assays have been described previously.^[^ ^33^ ^]^ 3000 cells per well were seeded and cultured in 24‐well ultralow‐attachment plates (3473, Corning, USA) with serum‐free DMEM/F12 media supplemented with B27 supplement (17504044, Gibco), N2 supplement (17502048, Gibco), 20 ng mL^−1^ recombinant human‐derived EGF (AF‐100‐15, PeproTech, USA) and 10 ng mL^−1^ bFGF (AF‐100‐18B, PeproTech) and cultured for 6–8 days (defined as P0). Tumorspheres with diameters larger than 50 µm were counted under a microscope. For tumorsphere passages, spheres were collected and dissociated into single cells by 0.1% trypsin and a gentle pipette and then filtered and replated to form a new generation sphere (defined as Px, x: passage number).

### Luciferase Reporter Assay

SOX2‐promoter luciferase plasmid was described previously.^[^ ^6^ ^]^ To generate enhancer luciferase reporter plasmids, the core DNA fragments of E1‐E4 and LIF‐promoter (−250 bp to +2000 bp) were subcloned into pGL3‐Baisc firefly luciferase plasmid (E1751, Promega, USA) and named as reporter fragments. Dual‐Luciferase Reporter Assay Kit (A1222, Promega) was used for luciferase reporter assay according to the manual. For measuring enhancer activity, 400 ng of firefly luciferase plasmids as well as 100 ng pRL‐NULL (E227A, Promega) *Renilla* luciferase plasmids were transfected into 1 × 10^5^ Cal27/Cal33/Fadu cells. Following 24‐h culture, cells were lysed and subjected to luminometers for *firefly* and *renilla* luciferase activity measurement. For detecting the TFs binding changes, cells were post‐transfected indicated plasmids or post‐treated with drugs/cytokines, then transfected with *firefly*/*renilla* luciferase plasmids and finally subjected to detect luciferase activity. Data were presented as the ratios of Firefly to *renilla* luciferase activity.

### Enzyme‐Linked Immunosorbent Assay (ELISA)

LIF in supernatant, cell/tissue lysis, and plasma were detected using human LIF ELISA Kits (#ELH097, FCMACS, China) according to the manufacturer's protocol. For tumor samples, a total weight of 100 mg tissue was homogenized in 1 mL lysis buffer and centrifuged at 12 000 rpm for 10 min to remove particles and polymers. For monolayer cells or tumorspheres, cells were routinely lysis and centrifuged. For blood samples, 2 mL of HNSCC patients` blood was centrifuged at 3000 rpm for 10 min at room temperature. The plasma was extracted and frozen at −80 °C until use.

### Patient‐Derived Cancer Cells (PDC) and Patient‐Derived Organoids (PDO) Model

Resection samples from patients with HNSCC were obtained from the Nanjing Medical University Affiliated Hospital of Stomatology with written informed consent from each patient and the Research Ethics Board approval in accordance with the Declaration of Helsinki. The surgically resected tumor samples were confirmed as HNSCC by two senior pathologists.

The detailed protocol for PDC generation has been reported previously.^[^ ^61^ ^]^

For PDO generation, fresh samples were transferred to cold advanced DMEM/F12 medium (12634010, Gibco), washed twice, cut into small pieces (1–3 mm^3^), and then digested at 37 °C in an enzyme solution (2 mg mL^−1^ collagenase I, 1 mg mL^−1^ DNase I, 200 U mL^−1^ hyaluronidase) for 40–60 min. Then, the digestion solution was neutralized, filtered by 100‐µm cell strainer, and centrifugated. Cell precipitation was washed by cold advanced DMEM/F12 medium and resuspended in 70% Matrigel Growth Factor Reduced (GFR) Basement Membrane Matrix (354230, Corning, USA). Cell‐mixed Matrigel was seeded into a pre‐heated 24‐well plate and incubated for 30 min at 37 °C before adding organoid culture medium. The culture medium was routinely changed every 2–3 days and the organoids were routinely passaged every 10–14 days. For passaging, the organoids were collected and resuspended in TrypLE (12604013, Gbico) at 37 °C for 10–20 min. Then, DMEM/F12 supplemented with 2–4% FBS was added to quench TrypLE activity after the organoids were digested into single cells. Cells were subsequently resuspended in 70% Matrigel and plated into a new 24‐well plate at suitable ratios (1:2 to 1:10). The detailed HNSCC‐specific PDO culture medium formulation is listed in Table S8 (Supporting Information).

### HNSCC Xenograft Model and Patient‐Derived Xenografts (PDX) Model

All animal experiments were complied with animal ethics guidelines and approved by the Institutional Animal Care and Use Committee of Nanjing Medical University. The detailed protocol for HNSCC xenograft and PDX models has been described previously.^[^ ^62^ ^]^

For limited dilution and tumorigenic assays, stable lentivirus‐transduced Fadu cells were trypsinized, counted, and diluted into an equal number of numbers (10^6^, 10^5^, 10^4^, 10^3^). These cells, either alone or in combination with an equivalent number of CAFs were inoculated subcutaneously on the left flank of nude mice. The longest and shortest diameters of each tumor were measured once every 3 days and the volume of tumor masses was estimated by the following equation: Volume = (the longest diameter) × (the shortest diameter)^2^/2.

To evaluate the therapeutic effect of the drug on xenograft modes, 1 × 10^6^ Fadu cells (along or admixed with an equal amount of CAFs) were injected into the flank of nude mice. Until the tumor size was ≈100 mm^3^, mice were randomly divided into two subgroups (≥6 animals per group) and received the following treatments: Stattic (4 mg kg^−1^, qod, i.p.), EC359 (5 mg kg^−1^, tiw, i.p.) or vehicle.

For PDX model generation, fresh HNSCC samples were thoroughly rinsed, trimmed into ≈1 mm^3,^ and transplanted into the left flank of 4–6 weeks female NOD/SCID mice. When the mass volume reached ≈100 mm^3^, mice were randomly divided into two groups and labeled. EC359 or vehicle (DMSO, control) was administered by intraperitoneal injection (5 mg kg^−1^, tiw, i.p., for 3 weeks) into tumor‐bearing mice.

### Immunohistochemical Staining (IHC) and Multiplex Immunohistochemistry (mIHC)

The protocol of IHC staining was conducted as previously described. In brief, paraformaldehyde‐fixed specimens were embedded and partitioned into 4 µm‐thick slides. Tissue sections were dewaxed, hydrated, and microwave‐treated with citrate for antigen retrieval. Then, sections were treated with 3% H_2_O_2_, blocked QuickBlock Blocking Buffer (P0220, Beyotime), and incubated with primary antibodies overnight at 4 °C. Second day, the slides were washed, incubated with secondary antibodies and followed by 3,3′‐Diaminobenzidine (DAB) and hematoxylin staining.

For mIHC assay, Multiplex IHC Detection kit (TSA amplification) (ab312827, Abcam, USA) was used according to the manual. In brief, routinely preparing tissue sections for IHC staining according to the abovementioned procedures. Sections were incubated with HRP‐labeled antibodies for 30 mins at room temperature and followed by tyramide signal amplification. Then, HRP was blocked and sections were incubated with next‐round HRP‐labeled antibody. These steps were repeated until all antibodies were labeled. Finally, tissues were counterstained with DAPI and fluorescent scanned by PerkinElmer Vectra system (PerkinElmer).

Relevant primary antibodies for IHC/mIHC are listed in Table S3 (Supporting Information).

### Flow Cytometry Analysis

Cells were trypsinized with EDTA‐free trypsin (15050065, Gbico), washed, and counted. Single‐cell suspension with equivalent cell numbers (1 × 10^6^) was incubated with CD133‐APC (0.5 µg per test) and CD44‐FITC (0.06 µg per test) according to the manufacturer's instructions and then analyzed by FACSCanto II (BD Bioscience). Relevant antibodies for flow cytometry analysis are listed in Table S3 (Supporting Information).

### Statistical Analysis

Quantitative data were reported as mean ± standard deviation (SD), derived mainly from no less than three independent experiments. Statistical analysis of the quantitative data was conducted using Student's *t*‐test (two‐tailed), Wilcoxon rank sum test (two‐tailed), or One‐way ANOVA, followed by post hoc testing with GraphPad Prism 9.0 software. The Chi‐squared and Fisher exact tests were employed to analyze circulating LIF levels and various clinicopathological parameters. Patient survival was assessed using the Kaplan‐Meier method and the Log‐rank test. Differences were supposed with statistical significance at ^#^ *p* ≥ 0.05, *p < 0.05, and **p < 0.01.

### Ethics Approval and Patient Consent Statement

The whole study was reviewed and approved by the Research Ethic Committee of Nanjing Medical University (Approval number: human samples collection/PDC/PDX: PJ2021‐030‐001; PDO: PJ2022‐061‐001; animal: IACUC‐1909025 and IACUC‐2103011). Written informed consent was obtained from patients before surgery with regard to clinical samples harvested for experiments. All animal experiments were complied with animal ethics guidelines and approved by Institutional Animal Care and Use Committee of Nanjing Medical University. Of note, the maximum tumor volume was permitted to not exceed 2000 mm^3^ for mice.

## Conflict of Interest

The authors declare no conflict of interest.

## Author Contributions

J.L., Y.W., and Z.W. contributed equally to this work. J.L., Y.W., and Z.W. performed all experiments, data collection, analyses, and manuscript writing. Y.W., P.D., and Y.W. took part in animal experiments and histological, and statistical analyses. D.W., H.J., and Y.W. performed patient inclusion, follow‐up, and data collection. J.C. conceived and supervised the whole project. All authors have read and approved the final manuscript.

## Supporting information

Supporting Information

## Data Availability

The data that support the findings of this study are available from the corresponding author upon reasonable request.
