# Supplementary material for: Super‐Enhancer Driven LIF/LIFR‐STAT3‐SOX2 Regulatory Feedback Loop Promotes Cancer Stemness in Head and Neck Squamous Cell Carcinoma
Source: Adv Sci (Weinh). 2024 Aug 29;11(40):2404476. doi: 10.1002/advs.202404476 (PMC11516160; doi:10.1002/advs.202404476)
Supplement: Supplementary file 1 — Supporting Information [file ADVS-11-2404476-s001.pdf]

ADVANCED SCIENCE
Open Access

Supporting Information

for *Adv. Sci.*, DOI 10.1002/advs.202404476

Super-Enhancer Driven LIF/LIFR-STAT3-SOX2 Regulatory Feedback Loop Promotes Cancer Stemness in Head and Neck Squamous Cell Carcinoma

*Jin Li, Yuhan Wang, Ziyu Wang, Yuxiang Wei, Pengfei Diao, Yaping Wu, Dongmiao Wang, Hongbing Jiang, Yanling Wang and Jie Cheng**

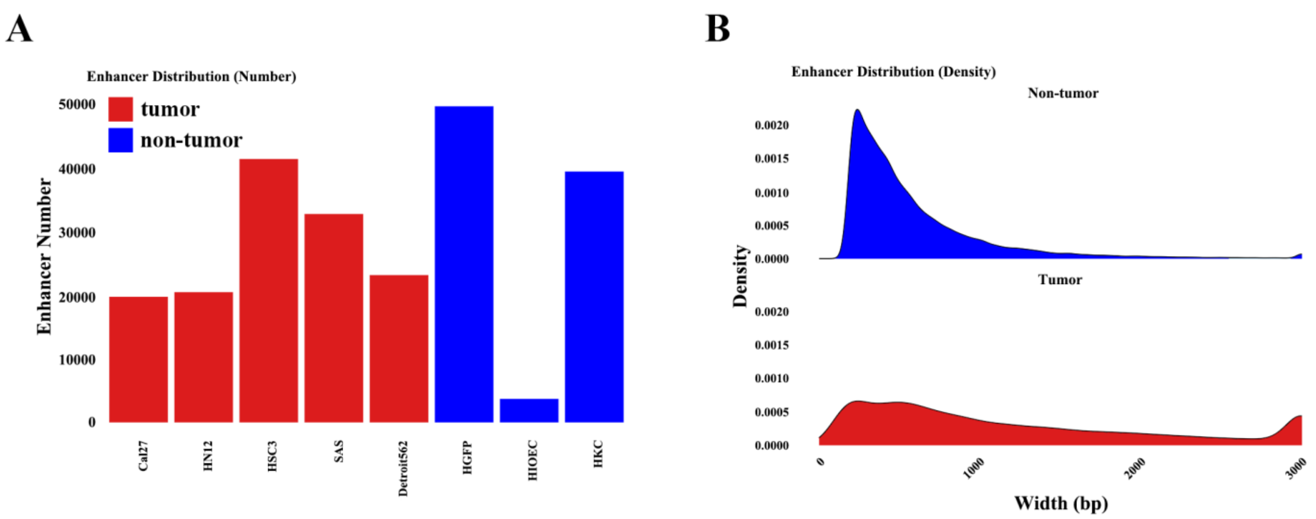

Supplementary Figure 1. (Related to Fig. 1B)

A. Enhancer numbers in 5 HNSCC cells and 3 non-tumor cells were displayed in the bar plot;

B. The density plots showed the enhancer length distribution in HNSCC and non-tumor cells.

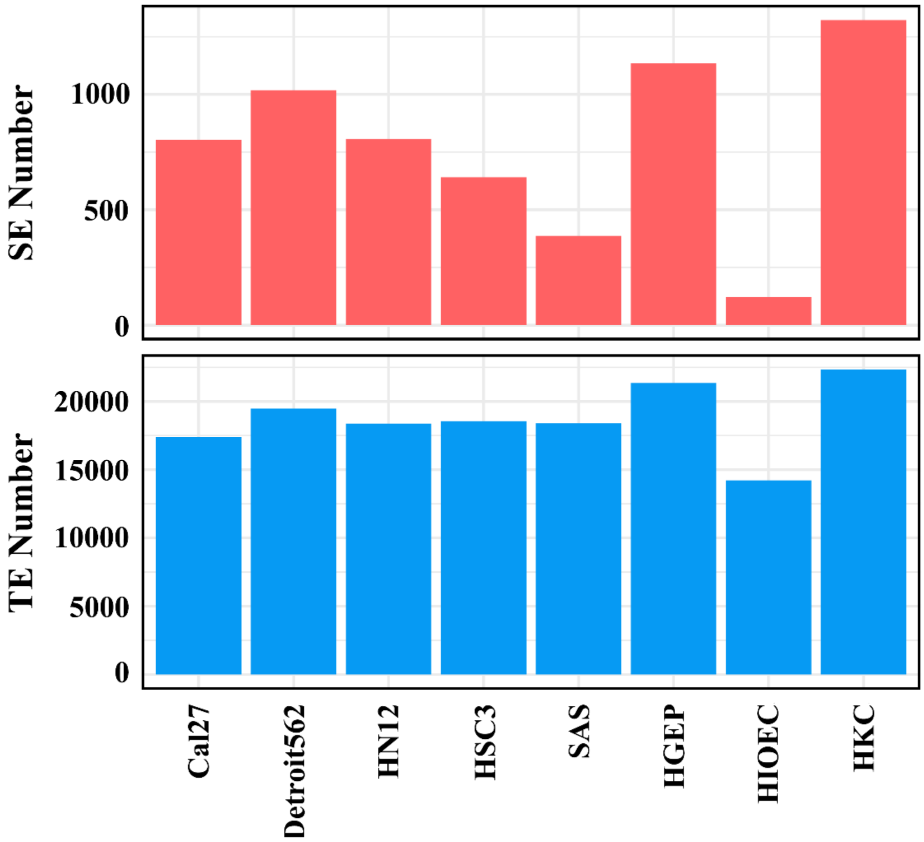

Supplementary Figure 2. (Related to Fig. 2A)

SE and TE numbers identified by the ROSE algorithm in 5 HNSCC cells and 3 non-tumor cells were shown.

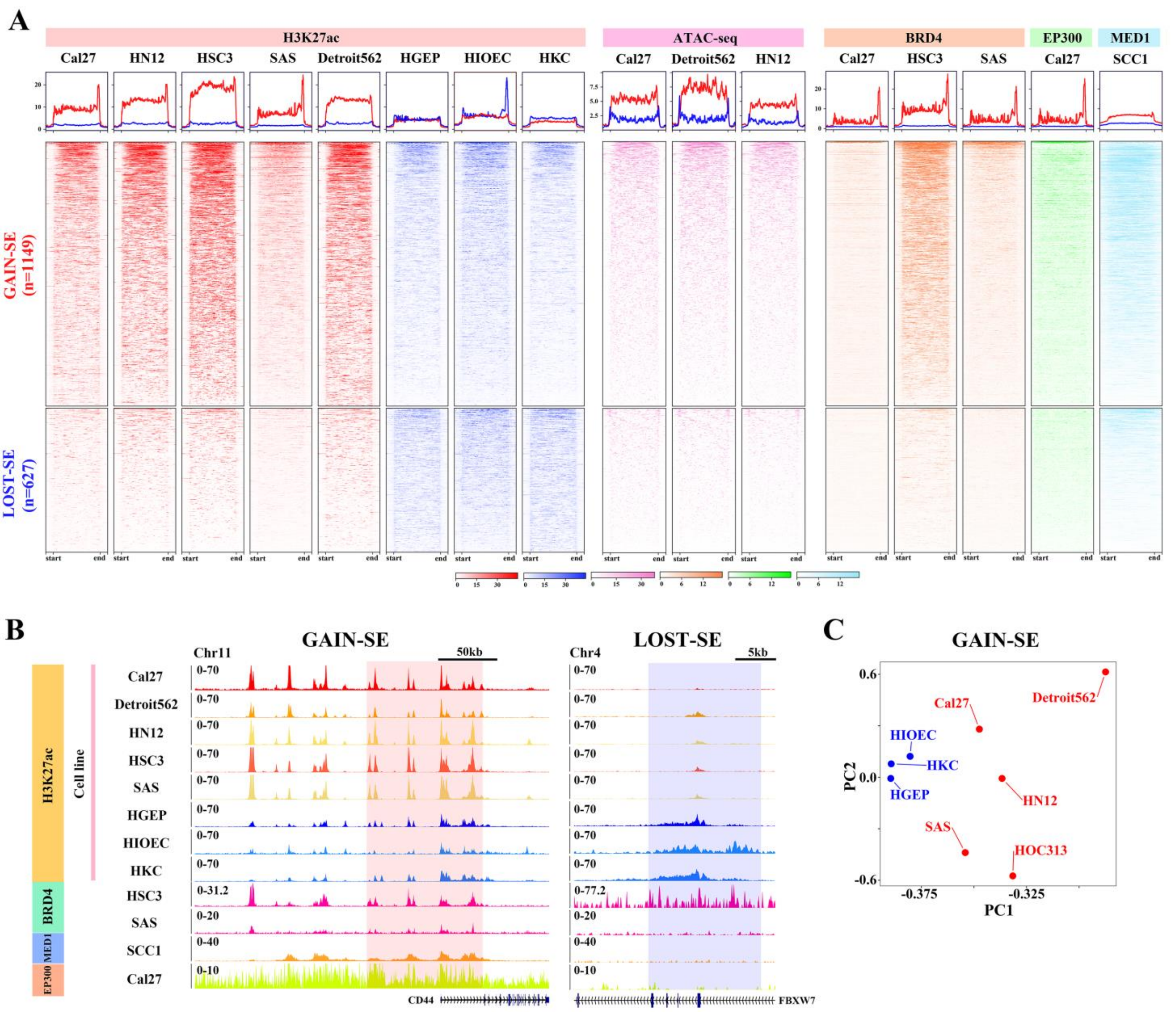

Supplementary Figure 3. Identify the variates SE in HNSCC

A. Heatmap plot displayed the signal enrichment of H3K27ac, ATAC-seq, BRD4, EP300 and MED1 in GAIN-SE and LOST-SE regions;

B. Genomic tracks plot displayed several representative GAIN-SEs and LOST-SE;

C. PCA analysis to classify HNSCC and non-tumor cells using the cumulative H3K27ac signal of GAIN-SE.

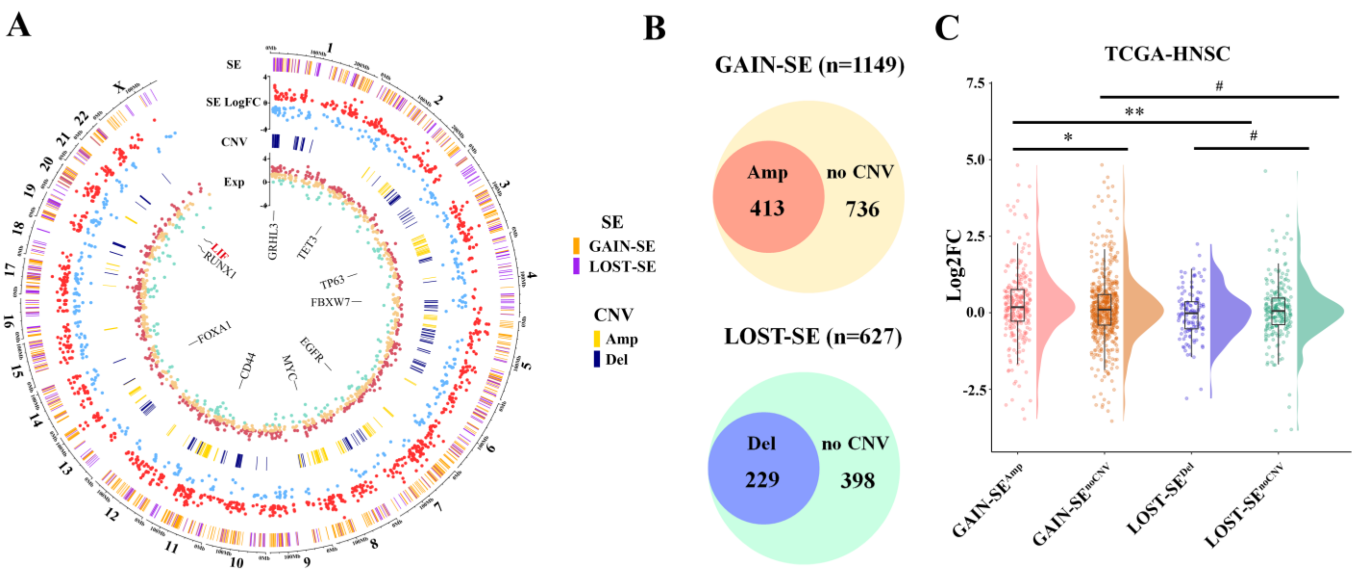

Supplementary Figure 4. CNV events on variate SEs in HNSCC

A. Circo plot displayed the genomic location and normalized fold change of H3K27ac signals (log2FC) of these variate SEs. CNV events and SE-associated gene mRNA level (log2(TPM+1)) retrieved from TCGA-HNSC dataset were displayed in outer ring;

B. Venn diagram showed the GAIN-SE or LOST-SE with CNV events;

C. GAIN-SE- or LOST-SE-associated gene expression changes (Log2-normalized) in TCGA-HNSC dataset was compared among 4 subgroups (GAIN-SE^{Amp}, GAIN-SE^{noCNV}, LOST-SE^{Del}, and LOST-SE^{noCNV}). Wilcoxon rank-sum test. $^{\#}P \geq 0.05$, $^{*}P < 0.05$, $^{**}P < 0.01$.

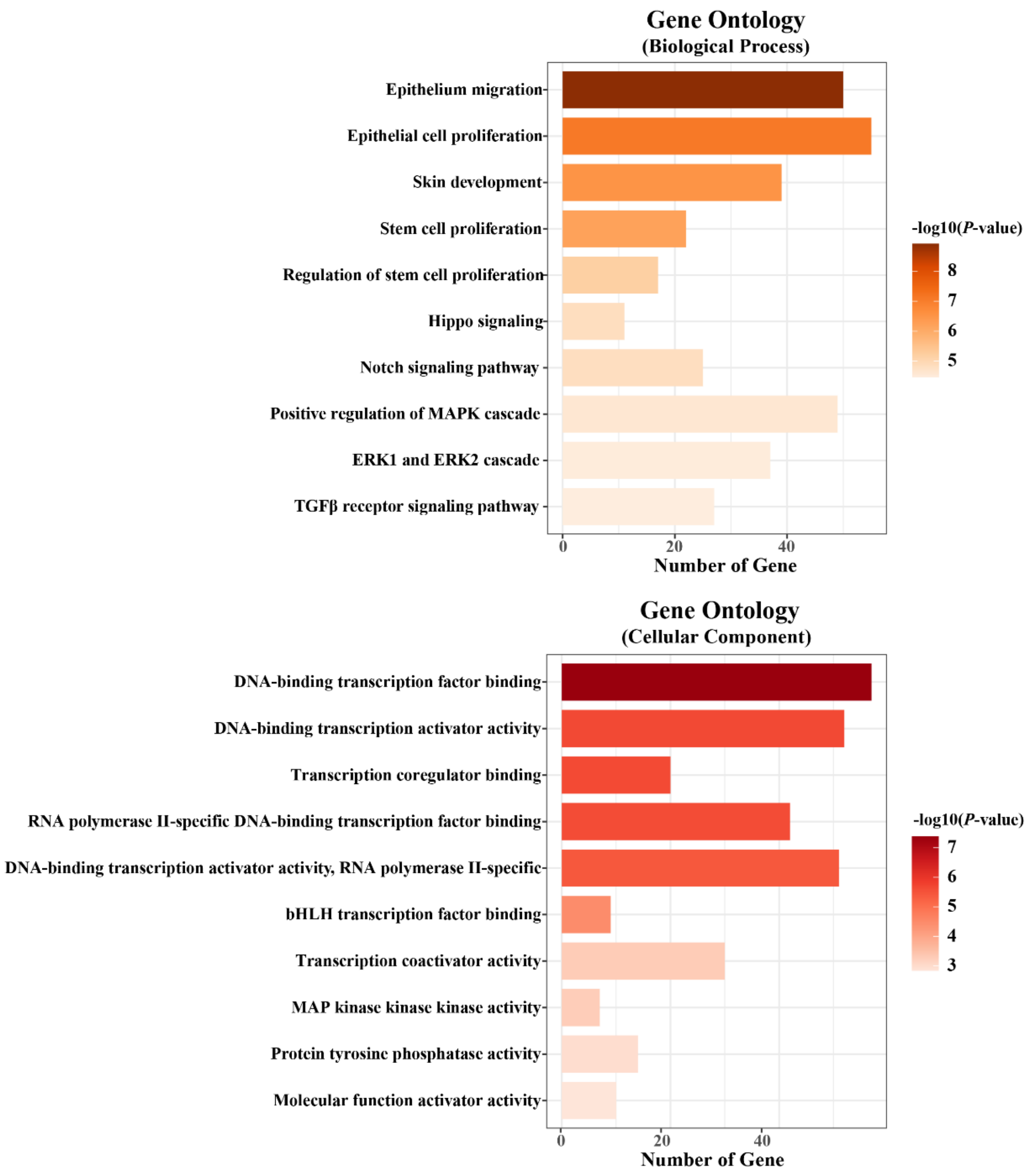

Supplementary Figure 5. (Related to Fig. 2H) GO analyses of GAIN-SE-associated genes

GO-BP and GO-CC analyses of GAIN-SE-associated genes.

Homer known Motif Analysis
(GAIN-SE)

Rank	Motif	TFs	*P*-value
1	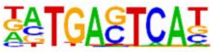	**BATF(bZIP)**<br>Th17-BATF-ChIP-Seq(GSE39756)	1×10^{-6}
2	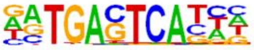	**Atf3(bZIP)**<br>GBM-ATF3-ChIP-Seq(GSE33912)	1×10^{-5}
3	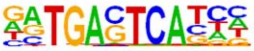	**Jun-AP1(bZIP)**<br>K562-cJun-ChIP-Seq(GSE31477)	1×10^{-5}
4	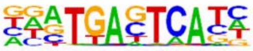	**Fra1(bZIP)**<br>BT549-Fra1-ChIP-Seq(GSE46166)	1×10^{-4}
5	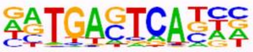	**Fosl2(bZIP)**<br>3T3L1-Fosl2-ChIP-Seq(GSE56872)	1×10^{-4}
6	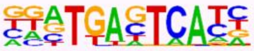	**Fos(bZIP)**<br>TSC-Fos-ChIP-Seq(GSE110950)	1×10^{-4}
7	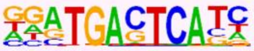	**Fra2(bZIP)**<br>Striatum-Fra2-ChIP-Seq(GSE43429)	1×10^{-4}
8	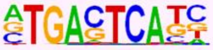	**AP-1(bZIP)**<br>ThioMac-PU.1-ChIP-Seq(GSE21512)	1×10^{-4}
9	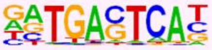	**JunB(bZIP)**<br>DendriticCells-Junb-ChIP-Seq(GSE36099)	1×10^{-4}
10	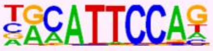	**TEAD3(TEA)**<br>HepG2-TEAD3-ChIP-Seq(Encode)	1×10^{-2}

Supplementary Figure 6. (Related to Fig. 2I)

Known motif enrichment on GAIN-SE regions was calculated by HOMER algorithm.

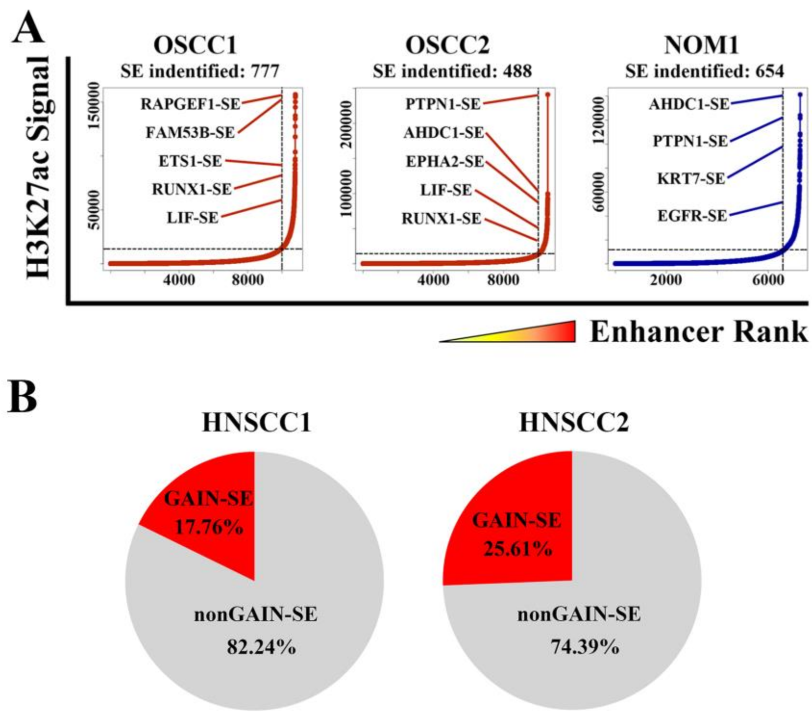

Supplementary Figure 7. Identification of SEs in primary HNSCC samples

A. Enhancers clusters in two primary HNSCC and one normal oral mucosa (NOM) samples ranked by summarized H3K27ac signals;

B. Pie chart showed the percentage of GAIN-SEs in the SE profiles of two HNSCC samples.

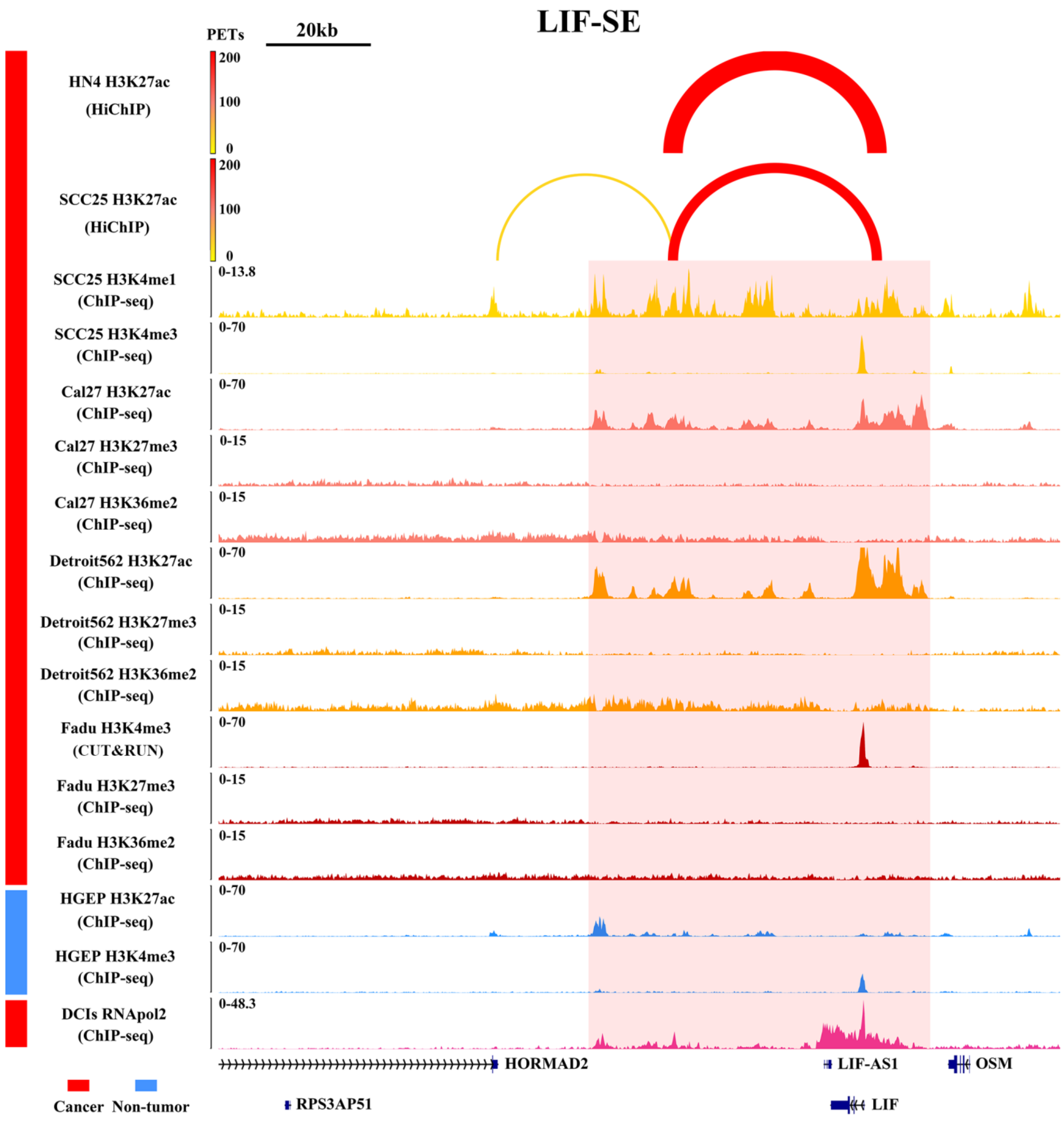

Supplementary Figure 8. (Related to Fig. 3A) The epigenetic signature on LIF-SE regions

Genomic tracks plot displayed the H3K27ac HiChIP loops (HN4 and SCC25), histone modifications (H3K27ac, H3K4me1, H3K4me3, H3K27me3, and H3K36me2) as well as RNApol2 enrichment on LIF-SE in the indicated cells.

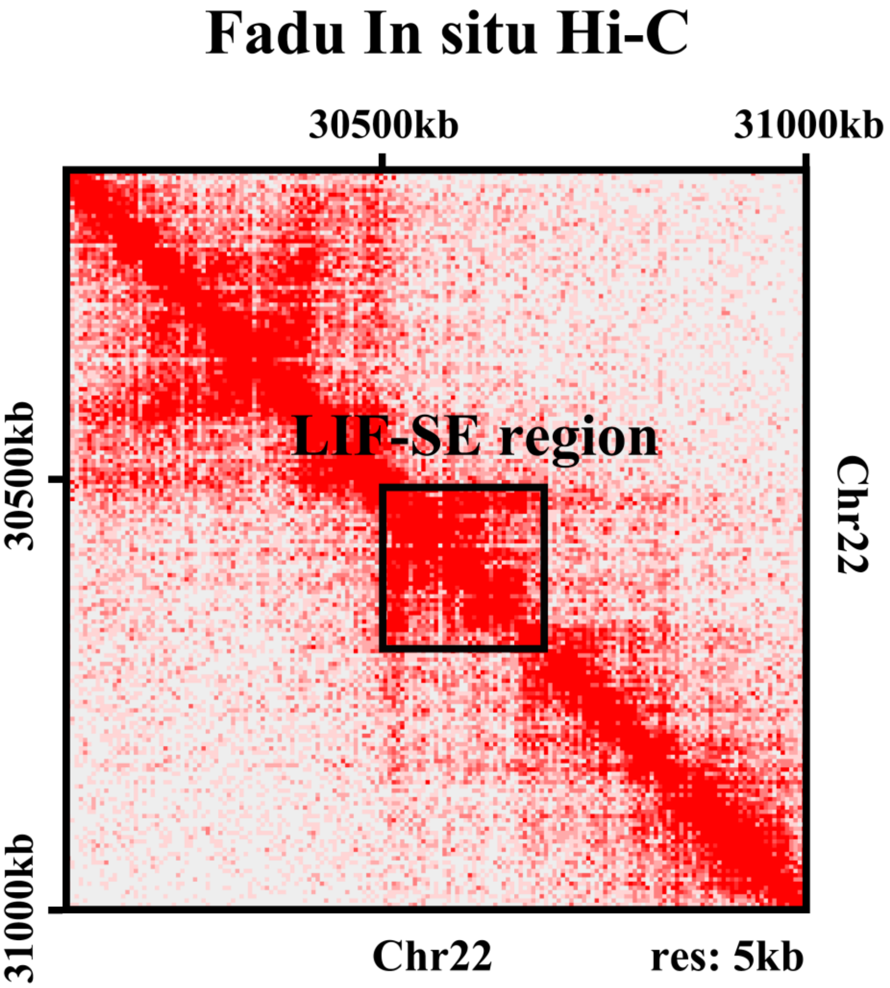

Supplementary Figure 9. (Related to Fig. 3A)

Fadu in situ Hi-C contact map showed the high 3D gnomic DNA contract frequency within LIF-SE regions.

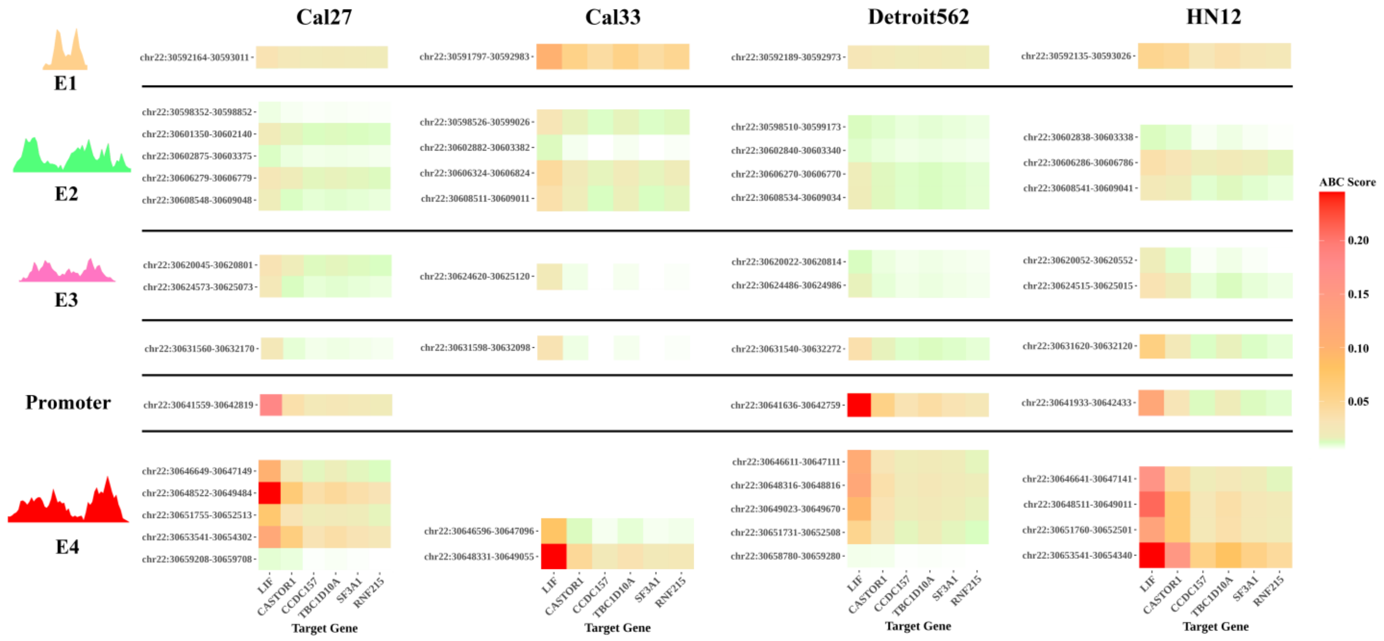

Supplementary Figure 10. (Related to Fig. 3B)

Heatmaps showed that the LIF promoter had the highest ABC scores compared with the promoters of other LIF-SE proximal genes.

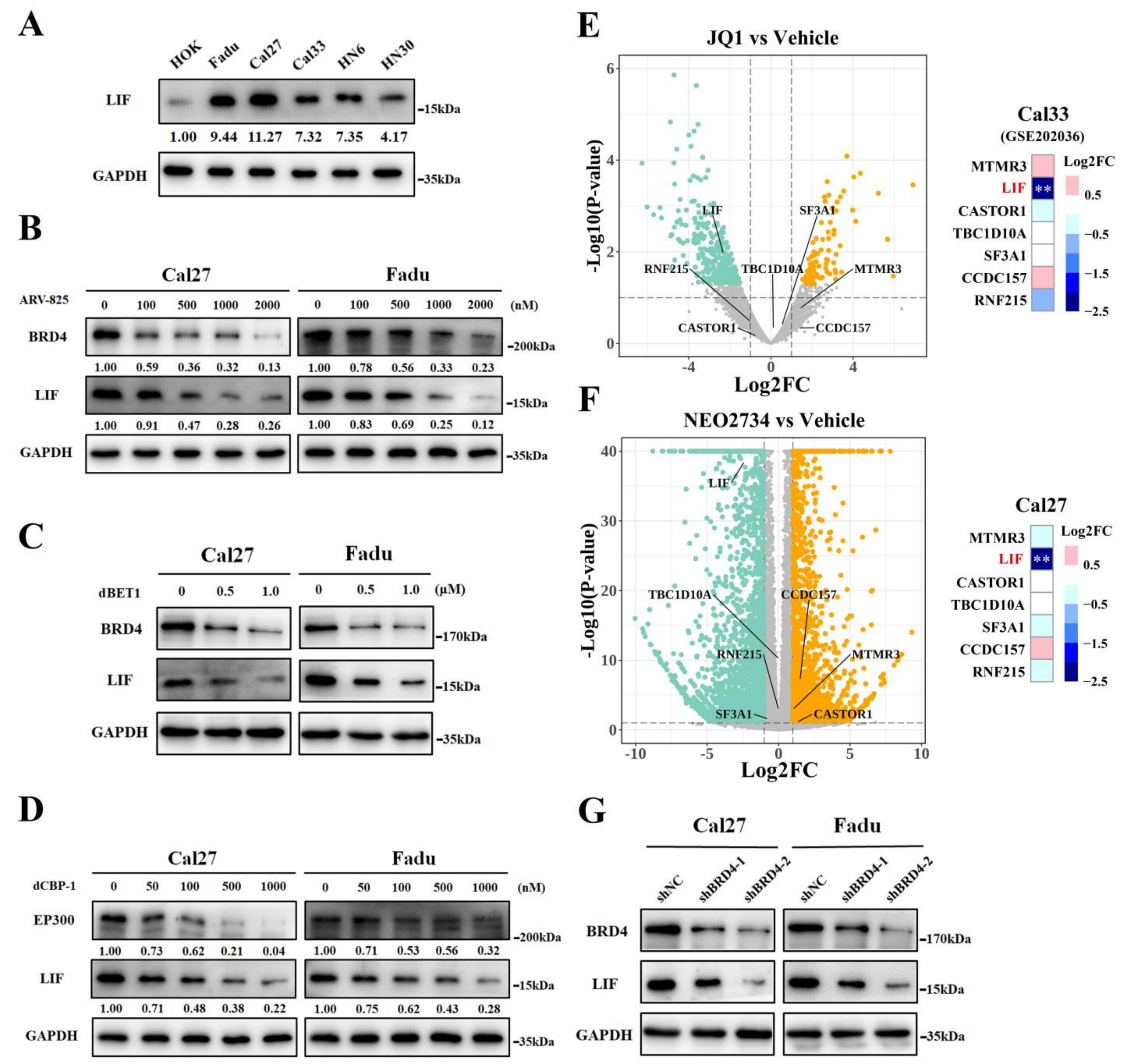

Supplementary Figure 11. (Related to Fig. 3C) Genetic manipulation, pharmacological inhibition/degeneration of SE-associated co-activators significantly reduces LIF expression.

A. Endogenous LIF protein expression was measured across multiple HNSCC cell lines as compared to normal oral epithelial (HOK);

B-D. Endogenous BRD4/EP300 and LIF protein abundance was measured in Cal27 and Fadu cells treated with the indicated concentration of PROTACs ARV-825 (0, 100, 500, 1000, 2000 nM, 24 hours, **B**), dBET1 (0, 0.5, 1.0 μM, 24 hours, **C**) or dCBP-1 (0, 50, 100, 500, 1000 nM, 24 hours. **D**);

E, F. Volcano plots showed the RNA-seq DEG expression levels in JQ1/DMSO-treated Cal33 cells

(GSE202036) or NEO2734/DMSO-treated Cal27 cells (left panel). Heatmaps displayed the log2FoldChange of the LIF-SE nearby genes. **FDR < 0.01;

G. Endogenous BRD4 and LIF protein abundance was measured in Cal27 and Fadu cells following BRD4 silencing.

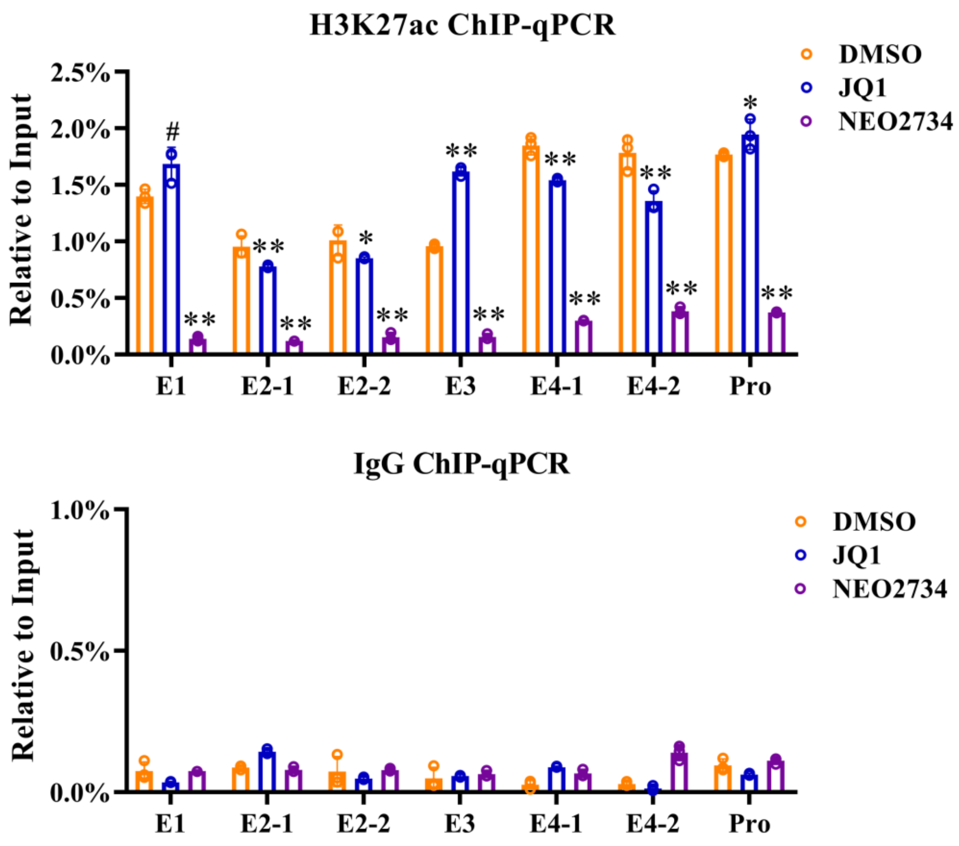

Supplementary Figure 12. (Related to Fig. 3H)

The H3K27ac/IgG binding on E1-E4 and LIF-promoter after JQ1 (1μM, 12h) or NEO2734 (500nM, 12h) exposure were measured by ChIP-qPCR. Student's *t* test. $^{\#}P \geq 0.05$, $^{*}P < 0.05$, $^{**}P < 0.01$.

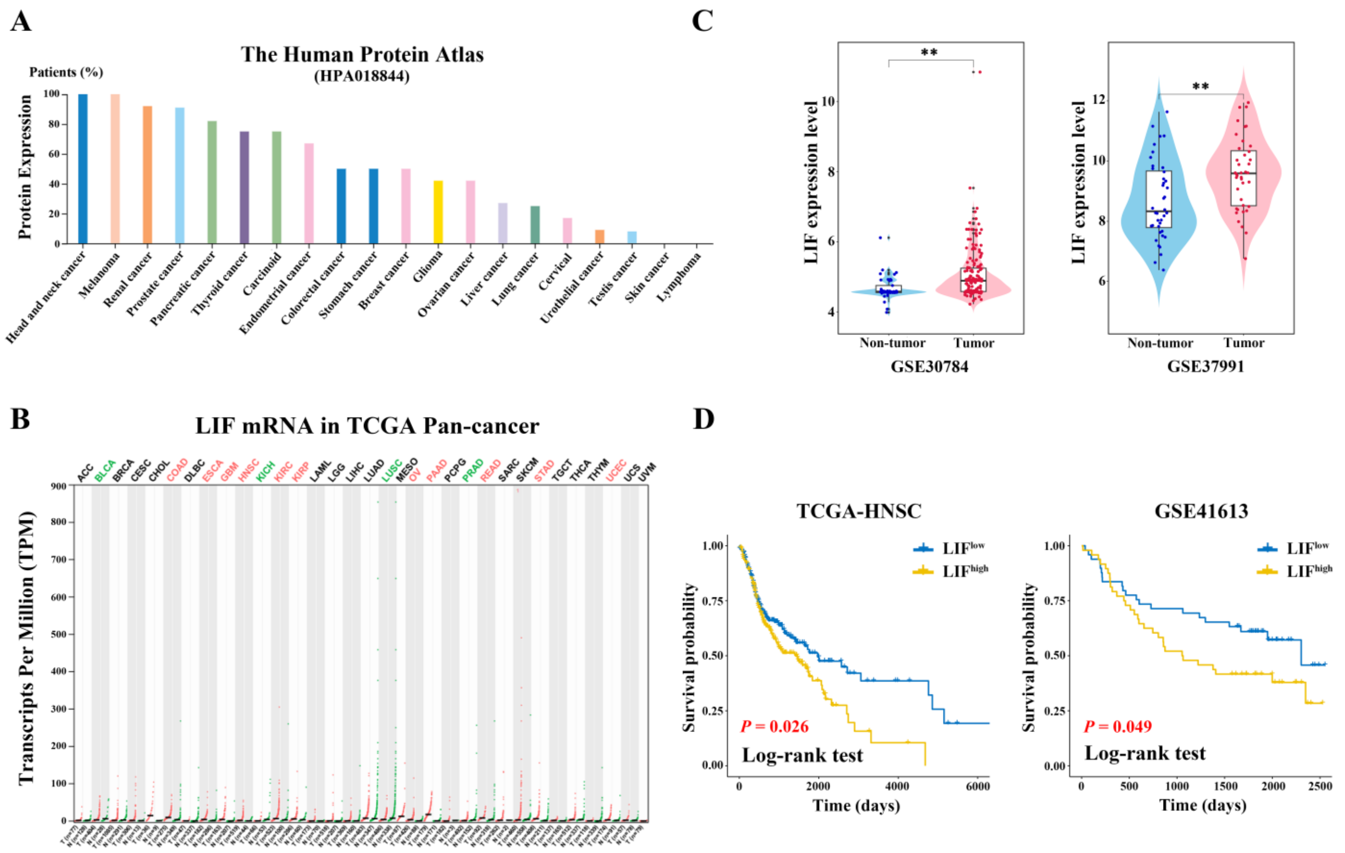

Supplementary Figure 13. LIF is overexpressed in HNSCC samples and associated with patient survival

A. LIF-positive IHC staining percentage in diverse human cancer sections was obtained from The Human Protein Atlas database;

B. The mRNA expression levels of LIF across various human cancers using the TCGA-PanCancer dataset were extracted from the GEPIA database. Wilcoxon rank-sum test;

C. LIF mRNA level in cancerous samples derived from GSE30784 and GSE37991 datasets was significantly overexpressed as compared to non-tumor counterparts. Wilcoxon rank-sum test, $**P < 0.01$;

D. Kaplan-Meier plots indicated that patients stratified with high expression of LIF (median-cutoff) had significantly lower overall survival rates than those with low expression LIF in the GSE41613 and the TCGA-HNSC datasets. Log-rank test.

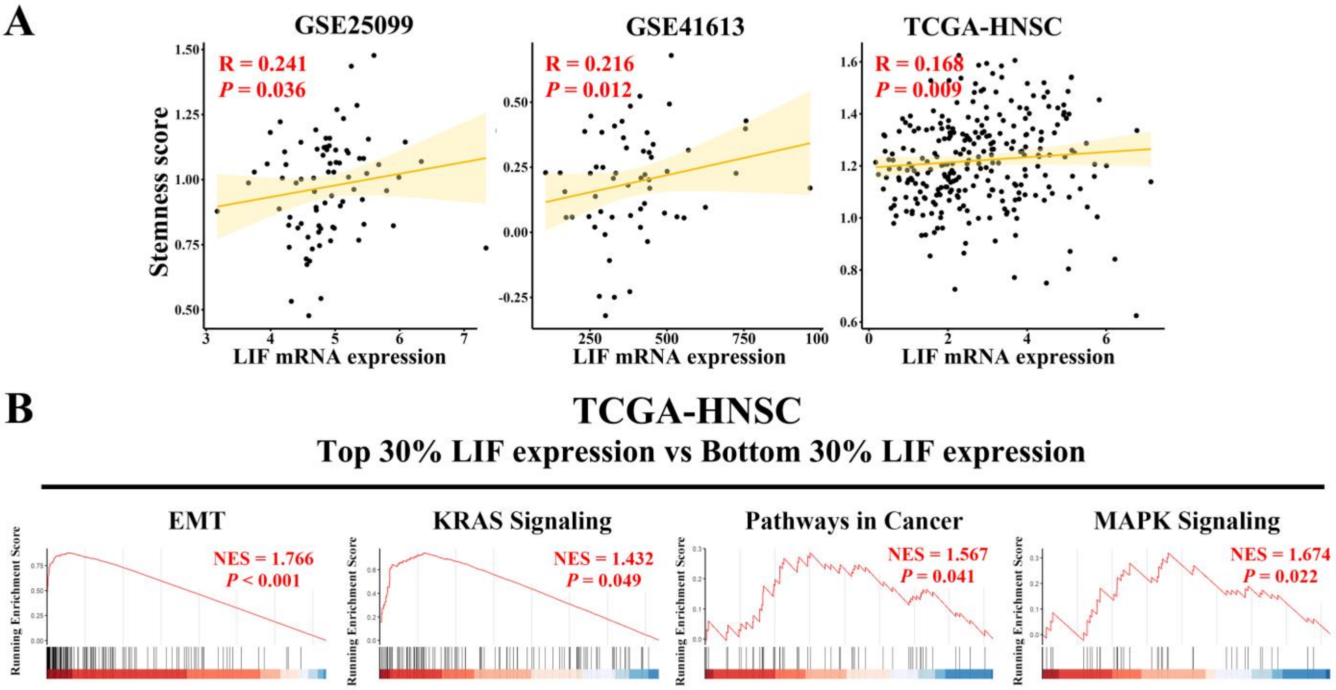

Supplementary Figure 14. LIF is positively correlated with CSC-related pathways.

A. The correlation between LIF mRNA and Stemness scores in three publicly available HNSCC datasets (GSE25099, GSE41613, and TCGA-HNSC) was assessed by the ssGSEA method. Spearman's correlation;

B. GSEA analysis from patients in the TCGA-HNSC dataset with the top 30% expression of LIF (n = 149) compared to those with the bottom 30% expression of LIF (n = 149) suggested that LIF-associated multiple oncogenic properties.

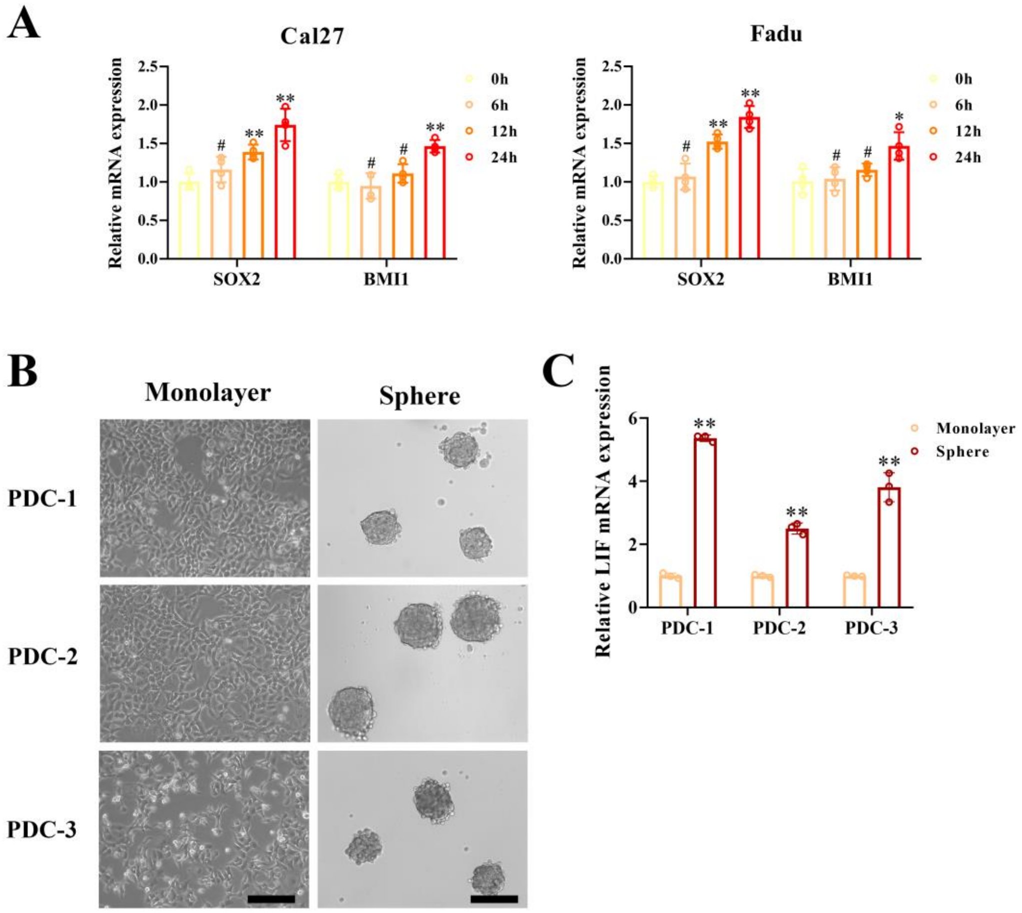

Supplementary Figure 15. (Related to Fig. 4A-B)

A. Cal27 and Fadu cells were treated with rhLIF (100 ng/mL) for different times (0, 6, 12, 24 h). Subsequently, SOX2 and BMI1 mRNA expressions were measured with qRT-PCR;

B, C. Representative morphology images (**B**) and LIF mRNA expression (**C**) of three different monolayer or spheroid cultured PDC cells were shown. Scale bar: 100 μm;

Data were presented as mean ± SD from 3 independent experiments. Student's *t* test. $^*P < 0.05$, $^{**}P < 0.01$.

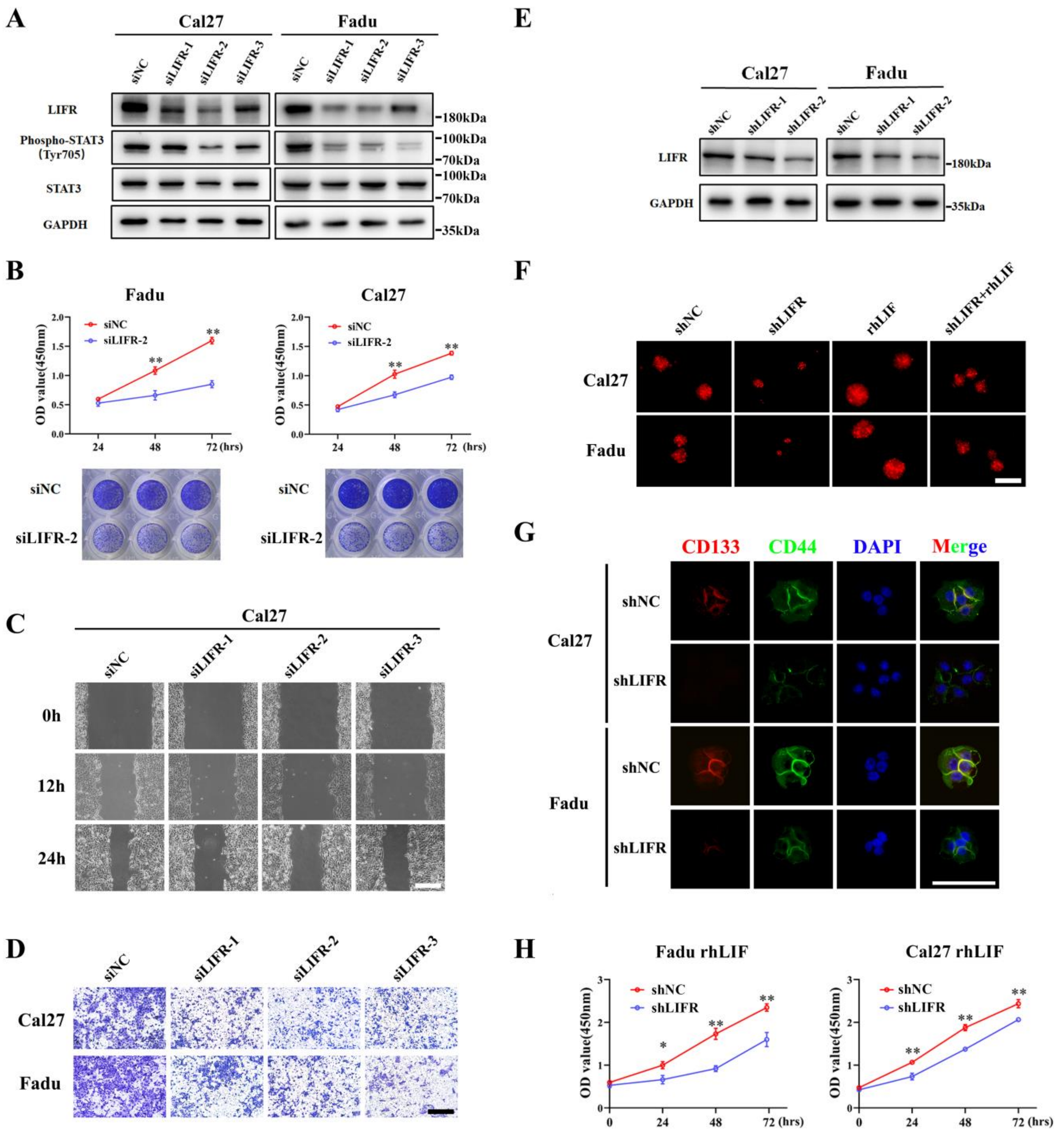

Supplementary Figure 16. LIFR silencing impairs tumor malignant phenotype in HNSCC cells

A. Protein abundance of LIFR and STAT3 phosphorylation in Fadu or Cal27 cells was measured after transfection with three independent human LIFR-targeting siRNAs for 72 hours using western blot assay;

B. Cell proliferation of Cal27 and Fadu cells was significantly reduced upon LIFR knockdown as measured by CCK-8 and crystal violet staining assays;

C, D. Cell migration of Cal27 (**C**) and invasion of Cal27 and Fadu (**D**) were remarkably impaired after LIFR silencing as measured by wound healing and Transwell assays. Scale bar: 100 μm;

E. Endogenous LIFR was efficiently reduced by shRNAs-targeting LIFR in Cal27 and Fadu cells;

F. Additional rhLIF supplementation had a minimal effect on pro-tumorsphere formation in LIFR-knockdown cells. Cal27 and Fadu cells were pre-labeled with DiI (red) when subjected to the sphere formation assay. Scale bar: 100 μm;

G. LIFR silencing significantly reduced CD44 and CD133 expression in HNSCC cells. CD44 (FTIC, green) and CD133 (Cy3, red) were determined by IF and the nucleus was counterstained with DAPI (blue). Scale bar: 100 μm;

H. Additional rhLIF supplementation could not restore the proliferative potential of LIFR-silenced Cal27 and Fadu cells as measured by the CCK-8 and crystal violet staining assays;

Data were presented as mean ± SD from 3 independent experiments. Student's t test. $*P < 0.05$, $**P < 0.01$.

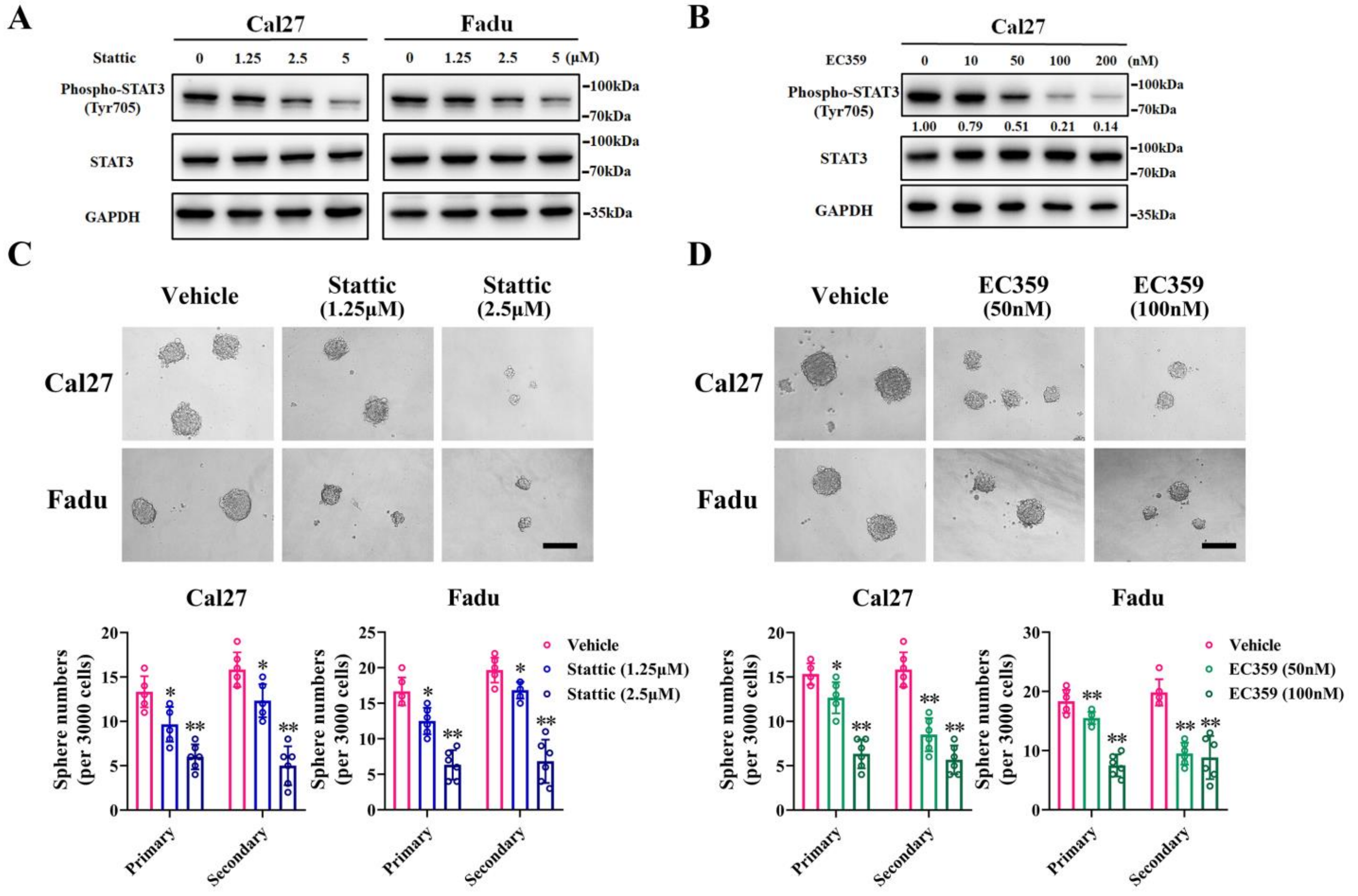

Supplementary Figure 17. Stattic/EC359 treatment inhibits STAT3 signaling activation and impairs tumorsphere formation in HNSCC cells.

A, B. The phosphorylated STAT3 ratio was significantly reduced after Stattic (**A**) or EC359 (**B**) treatment in HNSCC cells as detected via western blot assay;

C, D. Impaired tumorsphere formation was observed in cells treated with Stattic (**C**) or EC359 (**D**). Scale bar: 100 μm; Student's *t* test. **P* < 0.05, ***P* < 0.01.

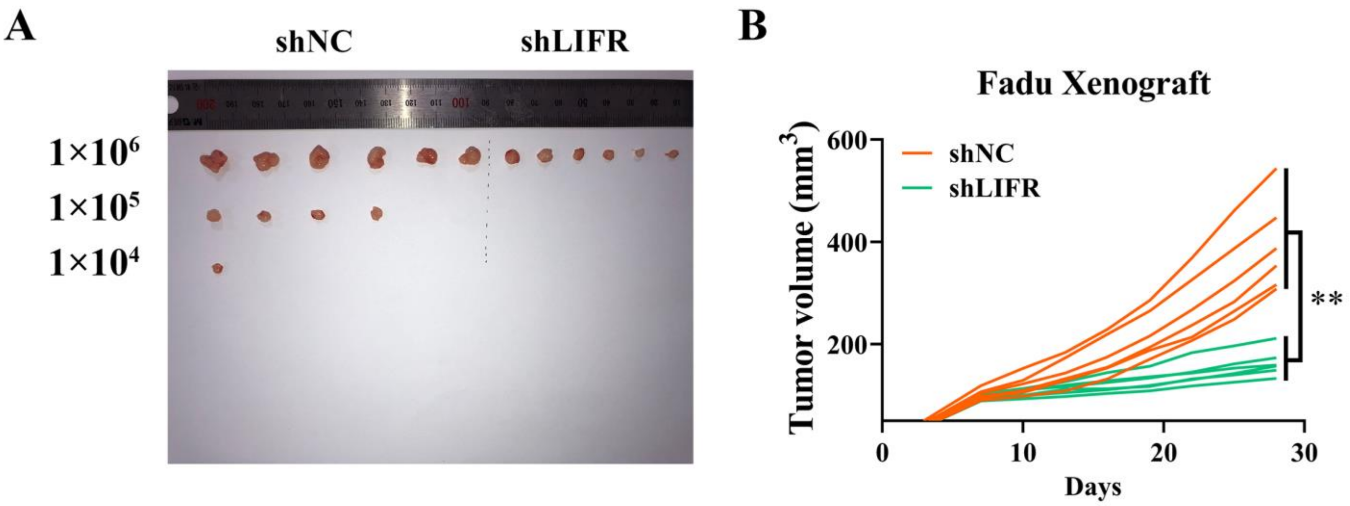

Supplementary Figure 18. (Related to Fig. 4I) LIFR knockdown impairs tumor growth *in vivo*.

A. Tumor-initiating capacities were determined with LIFR-silencing Fadu cells by limited dilution and tumorigenic assays *in vivo*. The original image for tumors (the first in vivo experiment) was shown;

B. Tumor volume was measured every three days after tumor initiation in mice inoculated with 10^6 cells; Student's *t* test. $^{**}P < 0.01$

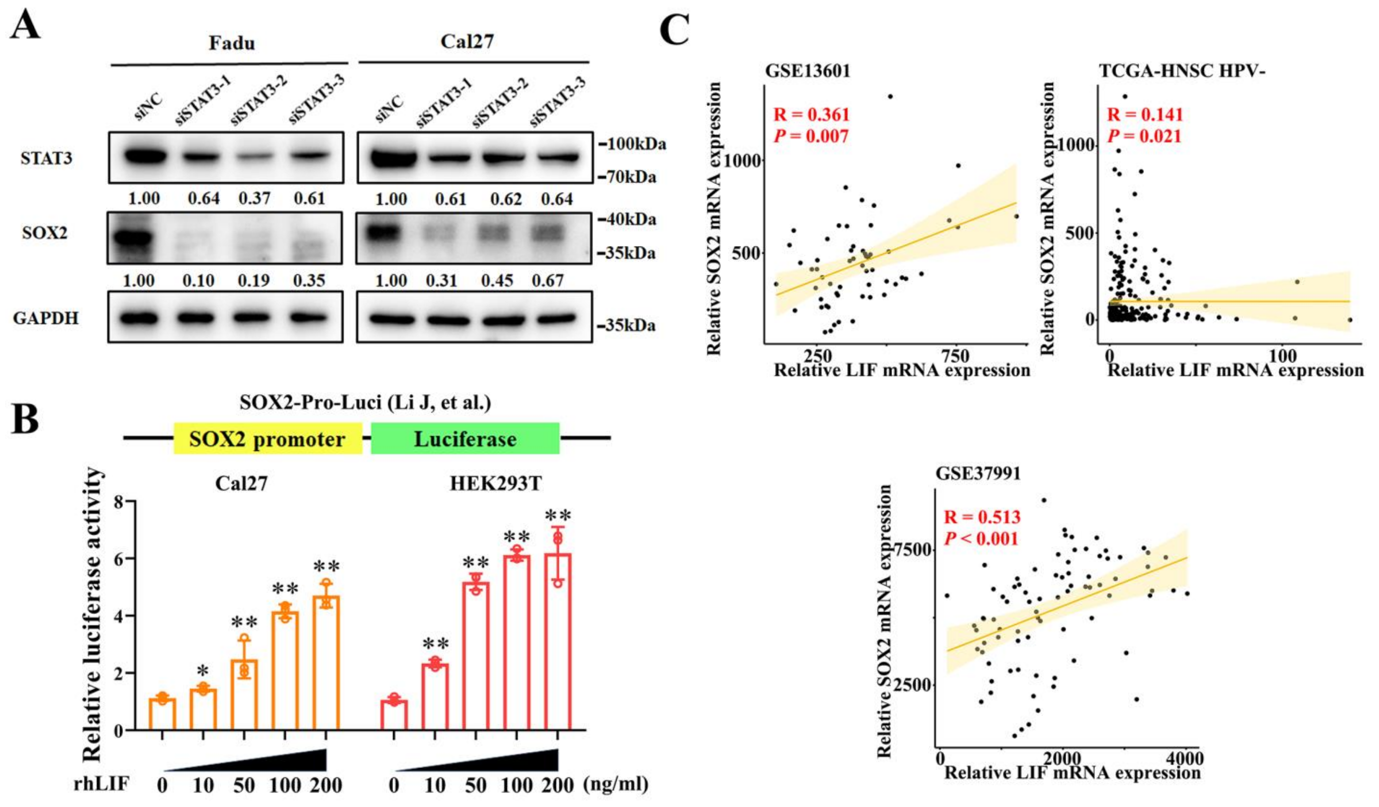

Supplementary Figure 19. LIF promotes SOX2 transcription via activating STAT3 signaling

A. The protein abundance of SOX2 was significantly reduced upon transfection of three independent human STAT3-targeting siRNAs in HNSCC cells;

B. Luciferase activities on SOX2 promoter were remarkably enhanced when HEK293T and Cal27 cells were exposed to increasing doses of rhLIF (0, 10, 50, 100, 200 ng/ml);

C. The correlation between SOX2 and LIF was assessed in the HNSCC datasets (GSE13601, TCGA-HNSC HPV- samples and GSE37991). Pearson's correlation.

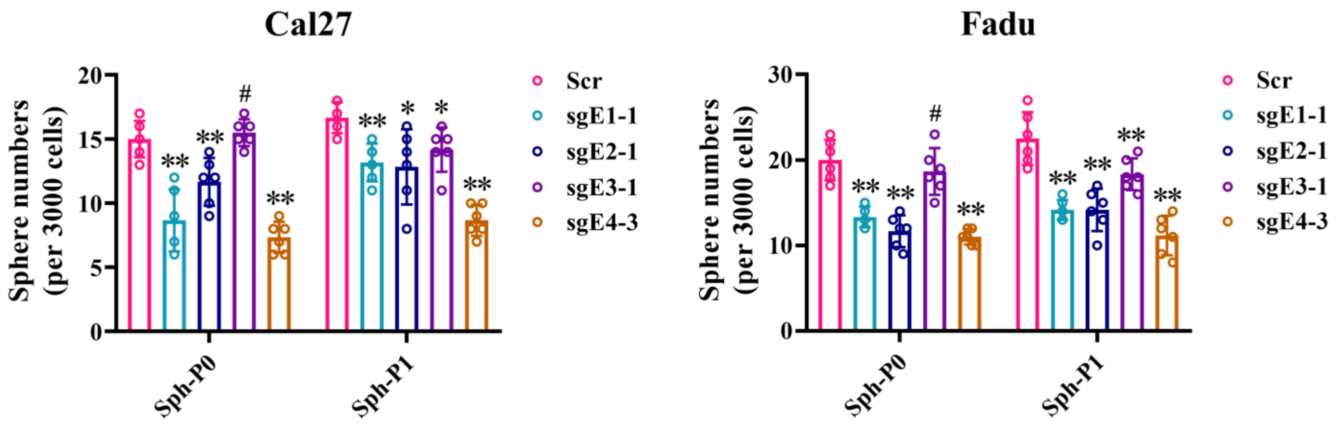

Supplementary Figure 20. (Related to Fig. 5A)

Impaired tumorsphere formation was observed upon E1, E2, and E4 repression by CRISPRi in HNSCC cells;

Data were presented as Mean ± SD. Student's *t* test. $^{\#}P \geq 0.05$, $*P < 0.05$, $**P < 0.01$.

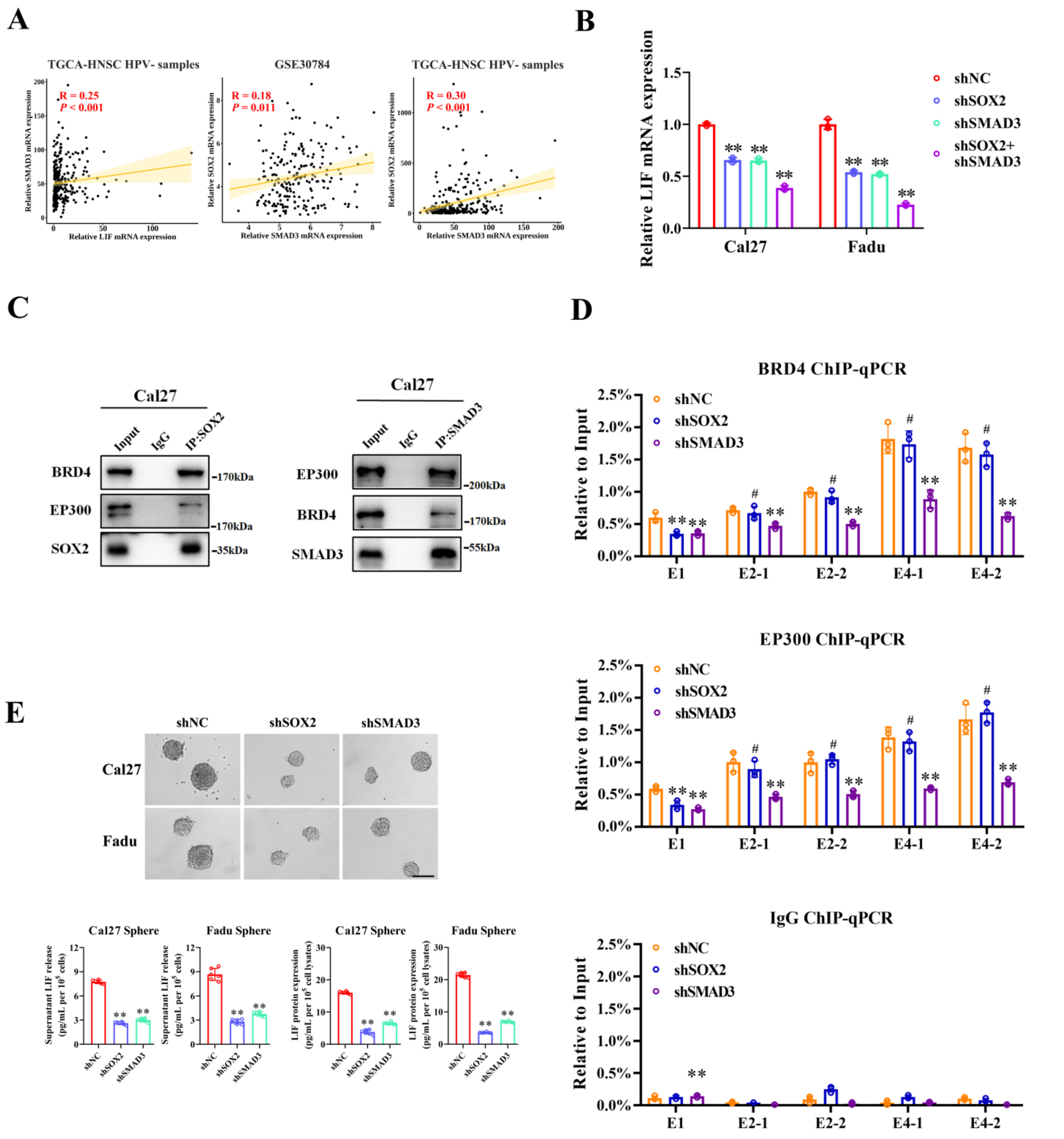

Supplementary Figure 21. SOX2/SMAD3 knockdown inhibits LIF transcription.

A. The correlations between SAMD3 and LIF/SOX2 mRNA were assessed in the HNSCC datasets (TCGA-HNSC HPV- samples, and GSE30784). Pearson's correlation;

B. LIF mRNA expression level in SOX2 or/and SMAD3 knockdown HNSCC cells was assessed by qRT-PCR;

C. SOX2/SMAD3 protein interacts with BRD4 and EP300 protein. Cal27 cell lysates were subjected to immunoprecipitation with anti-SOX2/anti-SMAD3 antibody followed by immunoblot with anti-EP300/anti-

BRD4 antibody;

D. The relative binding of BRD4, EP300 and IgG at E1, 2, 4 in SOX2/SMAD3-sliencing Cal27 and control cells were assessed by ChIP-qPCR;

E. Tumorsphere formation was determined in SOX2/SMAD3 silencing cells. The secreted LIF cytokine abundance in the supernatant (lower left panel) or the cellular LIF protein level in HNSCC cell lysate (lower right panel) was measured by ELISA;

Data were presented as Mean ± SD. Student's *t* test. $^{\#}P \geq 0.05$, $^{*}P < 0.05$, $^{**}P < 0.01$.

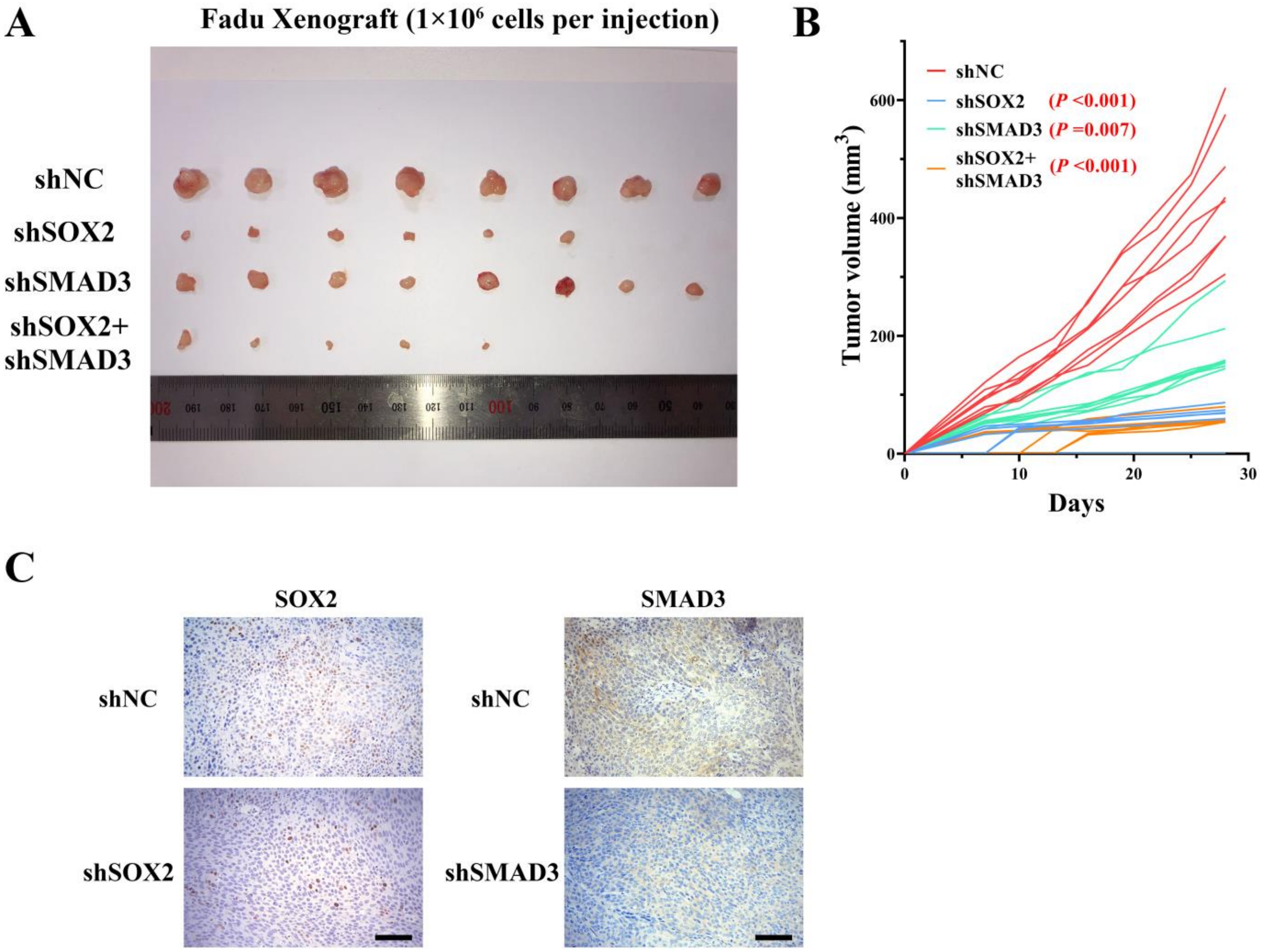

Supplementary Figure 22. (Related to Fig. 4I) SOX2/SMAD3 knockdown impairs tumor growth *in vivo*.

A, B. The image of Fadu xenograft in mice inoculated with 10^6 cells was displayed (**A**). Tumor volume was measured every three days after tumor initiation (**B**). Student's *t* test;

C. Representative SOX2 and SMAD3 immunohistochemical staining images of xenograft samples derived from Fadu xenografts with SOX2 or SMAD3 depletion. Scale bar: 100 μm.

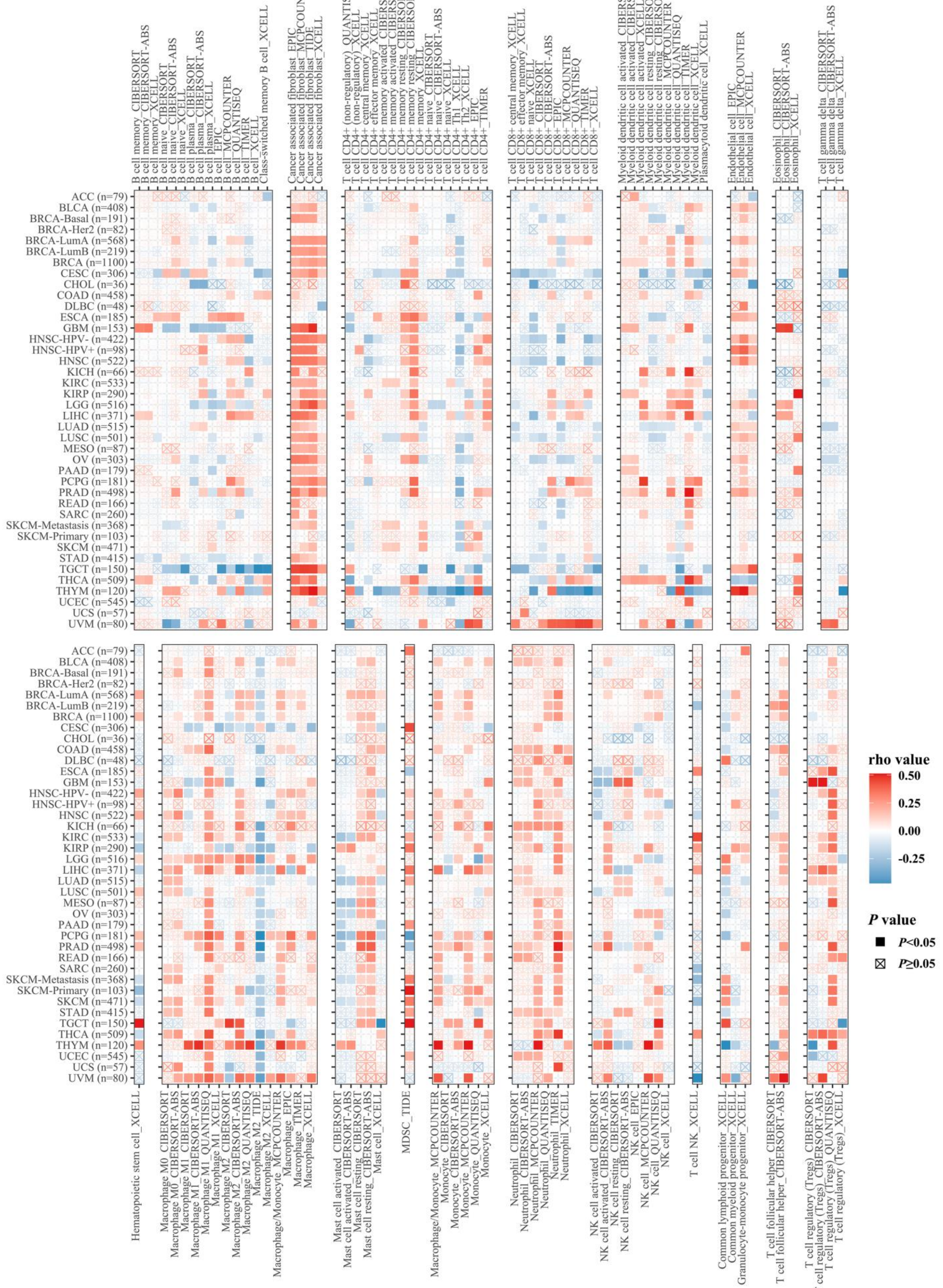
B cell memory_CIBERSORT
B cell memory_CIBERSORT-ABS
B cell memory_XCELL
B cell naive_CIBERSORT
B cell naive_CIBERSORT-ABS
B cell naive_XCELL
B cell plasma_CIBERSORT
B cell plasma_CIBERSORT-ABS
B cell plasma_XCELL
B cell_EPIC
B cell_MCPCOUNTER
B cell_QUANTISEQ
B cell_TIMER
B cell_XCELL
Class-switched memory B cell_XCELL
Cancer associated fibroblast_EPIC
Cancer associated fibroblast_MCPCOUNTER
Cancer associated fibroblast_TIDE
Cancer associated fibroblast_XCELL
T cell CD4+ (non-regulatory)_QUANTISEQ
T cell CD4+ (non-regulatory)_XCELL
T cell CD4+ central memory_XCELL
T cell CD4+ effector memory_XCELL
T cell CD4+ memory activated_CIBERSORT
T cell CD4+ memory activated_CIBERSORT-ABS
T cell CD4+ memory resting_CIBERSORT
T cell CD4+ memory resting_CIBERSORT-ABS
T cell CD4+ memory_XCELL
T cell CD4+ naive_CIBERSORT
T cell CD4+ naive_CIBERSORT-ABS
T cell CD4+ naive_XCELL
T cell CD4+ Th1_XCELL
T cell CD4+ Th2_XCELL
T cell CD4+_EPIC
T cell CD4+_TIMER
T cell CD8+ central memory_XCELL
T cell CD8+ effector memory_XCELL
T cell CD8+ naive_XCELL
T cell CD8+_CIBERSORT
T cell CD8+_CIBERSORT-ABS
T cell CD8+_EPIC
T cell CD8+_MCPCOUNTER
T cell CD8+_QUANTISEQ
T cell CD8+_TIMER
T cell CD8+_XCELL
Myeloid dendritic cell activated_CIBERSORT
Myeloid dendritic cell activated_CIBERSORT-ABS
Myeloid dendritic cell activated_XCELL
Myeloid dendritic cell resting_CIBERSORT
Myeloid dendritic cell resting_CIBERSORT-ABS
Myeloid dendritic cell_MCPCOUNTER
Myeloid dendritic cell_QUANTISEQ
Myeloid dendritic cell_TIMER
Myeloid dendritic cell_XCELL
Plasmacytoid dendritic cell_XCELL
Endothelial cell_EPIC
Endothelial cell_MCPCOUNTER
Endothelial cell_XCELL
Eosinophil_CIBERSORT
Eosinophil_CIBERSORT-ABS
Eosinophil_XCELL
T cell gamma delta_CIBERSORT
T cell gamma delta_CIBERSORT-ABS
T cell gamma delta_XCELL
ACC (n=79)
BLCA (n=408)
BRCA-Basal (n=191)
BRCA-Her2 (n=82)
BRCA-LumA (n=568)
BRCA-LumB (n=219)
BRCA (n=1100)
CESC (n=306)
CHOL (n=36)
COAD (n=458)
DLBC (n=48)
ESCA (n=185)
GBM (n=153)
HNSC-HPV- (n=422)
HNSC-HPV+ (n=98)
HNSC (n=522)
KICH (n=66)
KIRC (n=533)
KIRP (n=290)
LGG (n=516)
LIHC (n=371)
LUAD (n=515)
LUSC (n=501)
MESO (n=87)
OV (n=303)
PAAD (n=179)
PCPG (n=181)
PRAD (n=498)
READ (n=166)
SARC (n=260)
SKCM-Metastasis (n=368)
SKCM-Primary (n=103)
SKCM (n=471)
STAD (n=415)
TGCT (n=150)
THCA (n=509)
THYM (n=120)
UCEC (n=545)
UCS (n=57)
UVM (n=80)
ACC (n=79)
BLCA (n=408)
BRCA-Basal (n=191)
BRCA-Her2 (n=82)
BRCA-LumA (n=568)
BRCA-LumB (n=219)
BRCA (n=1100)
CESC (n=306)
CHOL (n=36)
COAD (n=458)
DLBC (n=48)
ESCA (n=185)
GBM (n=153)
HNSC-HPV- (n=422)
HNSC-HPV+ (n=98)
HNSC (n=522)
KICH (n=66)
KIRC (n=533)
KIRP (n=290)
LGG (n=516)
LIHC (n=371)
LUAD (n=515)
LUSC (n=501)
MESO (n=87)
OV (n=303)
PAAD (n=179)
PCPG (n=181)
PRAD (n=498)
READ (n=166)
SARC (n=260)
SKCM-Metastasis (n=368)
SKCM-Primary (n=103)
SKCM (n=471)
STAD (n=415)
TGCT (n=150)
THCA (n=509)
THYM (n=120)
UCEC (n=545)
UCS (n=57)
UVM (n=80)
Hematopoietic stem cell_XCELL
Macrophage M0_CIBERSORT
Macrophage M0_CIBERSORT-ABS
Macrophage M1_CIBERSORT
Macrophage M1_CIBERSORT-ABS
Macrophage M1_QUANTISEQ
Macrophage M1_XCELL
Macrophage M2_CIBERSORT
Macrophage M2_CIBERSORT-ABS
Macrophage M2_QUANTISEQ
Macrophage M2_TIDE
Macrophage M2_XCELL
Macrophage/Monocyte_MCPCOUNTER
Macrophage_EPIC
Macrophage_TIMER
Macrophage_XCELL
Mast cell activated_CIBERSORT
Mast cell activated_CIBERSORT-ABS
Mast cell resting_CIBERSORT
Mast cell resting_CIBERSORT-ABS
Mast cell_XCELL
MDSC_TIDE
Macrophage/Monocyte_MCPCOUNTER
Monocyte_CIBERSORT
Monocyte_CIBERSORT-ABS
Monocyte_MCPCOUNTER
Monocyte_QUANTISEQ
Monocyte_XCELL
Neutrophil_CIBERSORT
Neutrophil_CIBERSORT-ABS
Neutrophil_MCPCOUNTER
Neutrophil_QUANTISEQ
Neutrophil_TIMER
Neutrophil_XCELL
NK cell activated_CIBERSORT
NK cell activated_CIBERSORT-ABS
NK cell resting_CIBERSORT
NK cell resting_CIBERSORT-ABS
NK cell_EPIC
NK cell_MCPCOUNTER
NK cell_QUANTISEQ
NK cell_XCELL
T cell NK_XCELL
Common lymphoid progenitor_XCELL
Common myeloid progenitor_XCELL
Granulocyte-monocyte progenitor_XCELL
T cell follicular helper_CIBERSORT
T cell follicular helper_CIBERSORT-ABS
T cell regulatory (Tregs)_CIBERSORT
T cell regulatory (Tregs)_CIBERSORT-ABS
T cell regulatory (Tregs)_QUANTISEQ
T cell regulatory (Tregs)_XCELL
rho value
0.50
0.25
0.00
-0.25
P value
P<0.05
P≥0.05

Supplementary Figure 23. LIF mRNA expression is positively associated with CAFs infiltration.

Heatmap illustrated the correlations between LIF mRNA expression and immune cells/CAFs infiltration in the TCGA-PanCancer dataset. The immune/CAFs infiltration scores were obtained from the TIMER2.0 database (http://timer.cistrome.org/). Spearman's correlation.

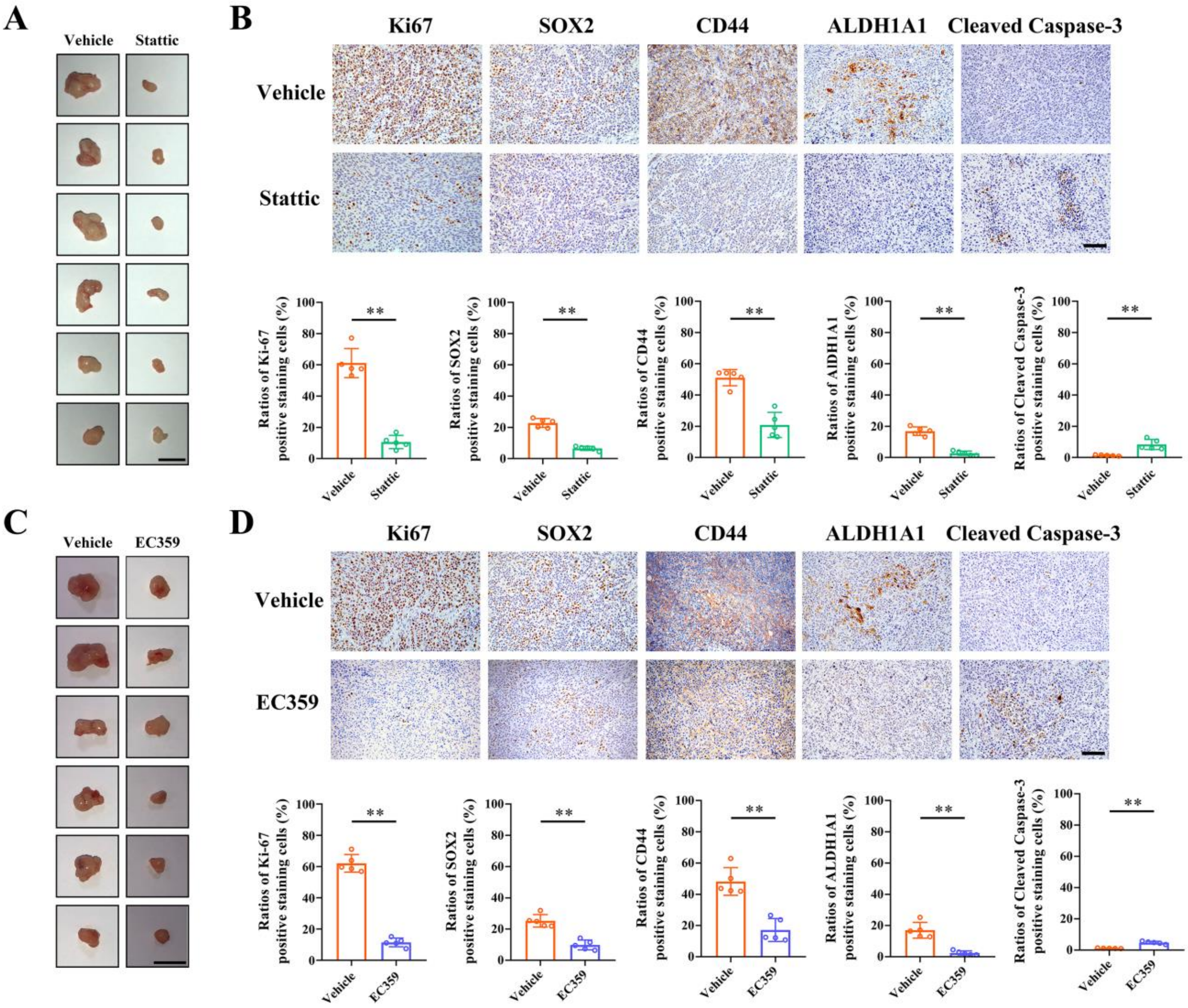

Supplementary Figure 24. (Related to Fig. 7A-D) Stattic or EC359 treatment impaired tumor growth and CSC subpopulations in Fadu xenograft model.

A-B. Representative tumor (**A**) and IHC staining images (**B**, upper panel) and corresponding quantification data (**B**, bottom panel) of Ki-67, Cleaved Casepase-3, and CSCs markers (including SOX2, CD44 and ALDH1A1) in Fadu derived xenograft samples treated with Stattic or vehicle were shown. Scale bar: 100 μm; **C-D.** Representative tumor (**C**) and IHC staining images (**D**, upper panel) and corresponding quantification data (**D**, bottom panel) of Ki-67, Cleaved Casepase-3, and CSCs markers (including SOX2, CD44 and ALDH1A1) in Fadu derived xenograft samples treated with EC359 or vehicle were shown. Scale bar: 100 μm; Data were presented as Mean ± SD. Student's *t* test. $**P < 0.01$.

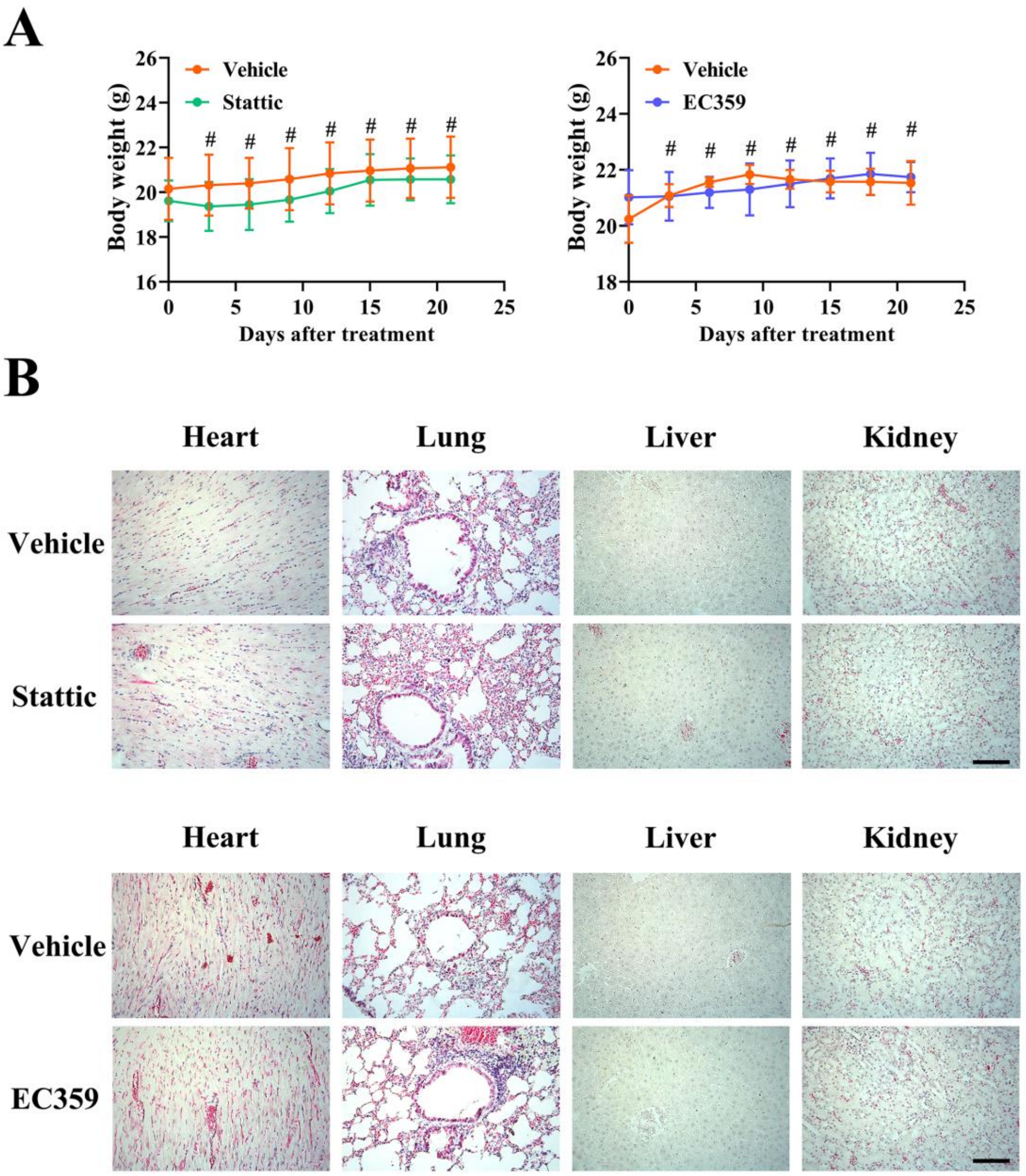

Supplementary Figure 25. (Related to Fig. 7A-D)

A. Stattic or EC359 treatment caused no significant loss of body weight in mice during the whole treatment;

B. Stattic or EC359 treatment caused no obvious abnormalities of vital organs including heart, lung, liver and kidney as measured by H&E staining. Scale bar: 100 μm.

Data were presented as Mean ± SD. Student's *t* test. $^{\#}P \geqslant 0.05$.

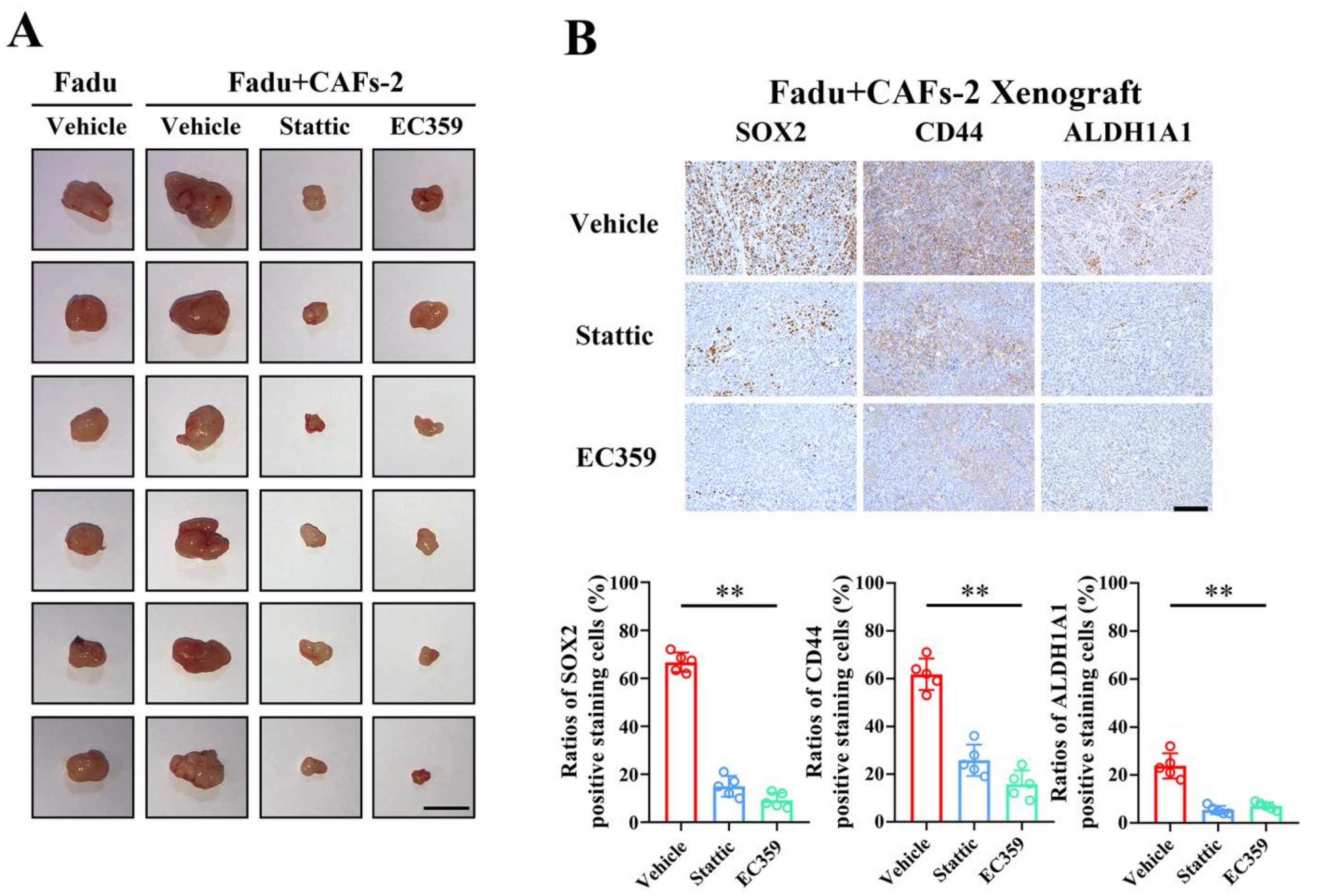

Supplementary Figure 26. (Related to Fig. 7F-G) Stattic or EC359 treatment impaired tumor growth and CSC subpopulations in CAFs-2 admixed Fadu xenograft model.

A-B. Representative tumor (**A**, scale bar: 1cm), IHC staining images (**B**, upper panel, scale bar: 100 μm) and corresponding quantification data (**B**, lower panel) of SOX2, CD44, and ALDH1A1 in CAFs admixed Fadu derived xenograft samples treated with Stattic, EC359 or vehicle were shown;

Data were presented as Mean ± SD. Student's *t* test. $^{**}P < 0.01$.

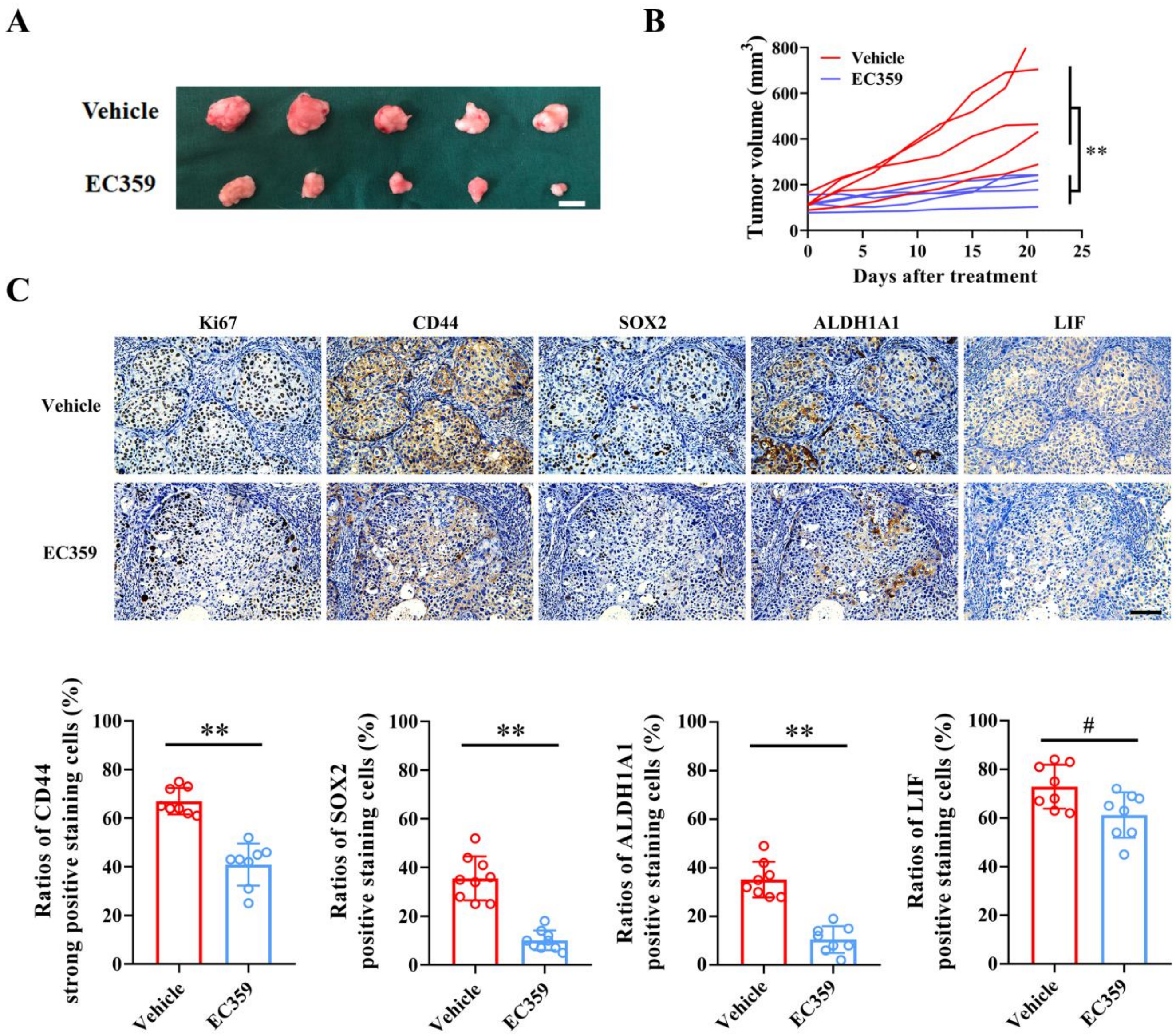

Supplementary Figure 27. EC359 inhibited tumor growth and CSC subpopulations in HNSCC PDX model.

A, B. The PDX masses image in EC359 (5 mg/kg, tiw, i.p.) or Vehicle treatment group was shown (**A**). Tumor volume was measured (**B**). Scale bar: 1cm.

C. Representative IHC staining images and corresponding quantification data of CD44, SOX2, ALDH1A1 and LIF in PDX samples treated with EC359 or vehicle were shown. Scale bar: 100μm.

Data were presented as Mean ± SD. Student's *t* test. $^{**}P < 0.01$.

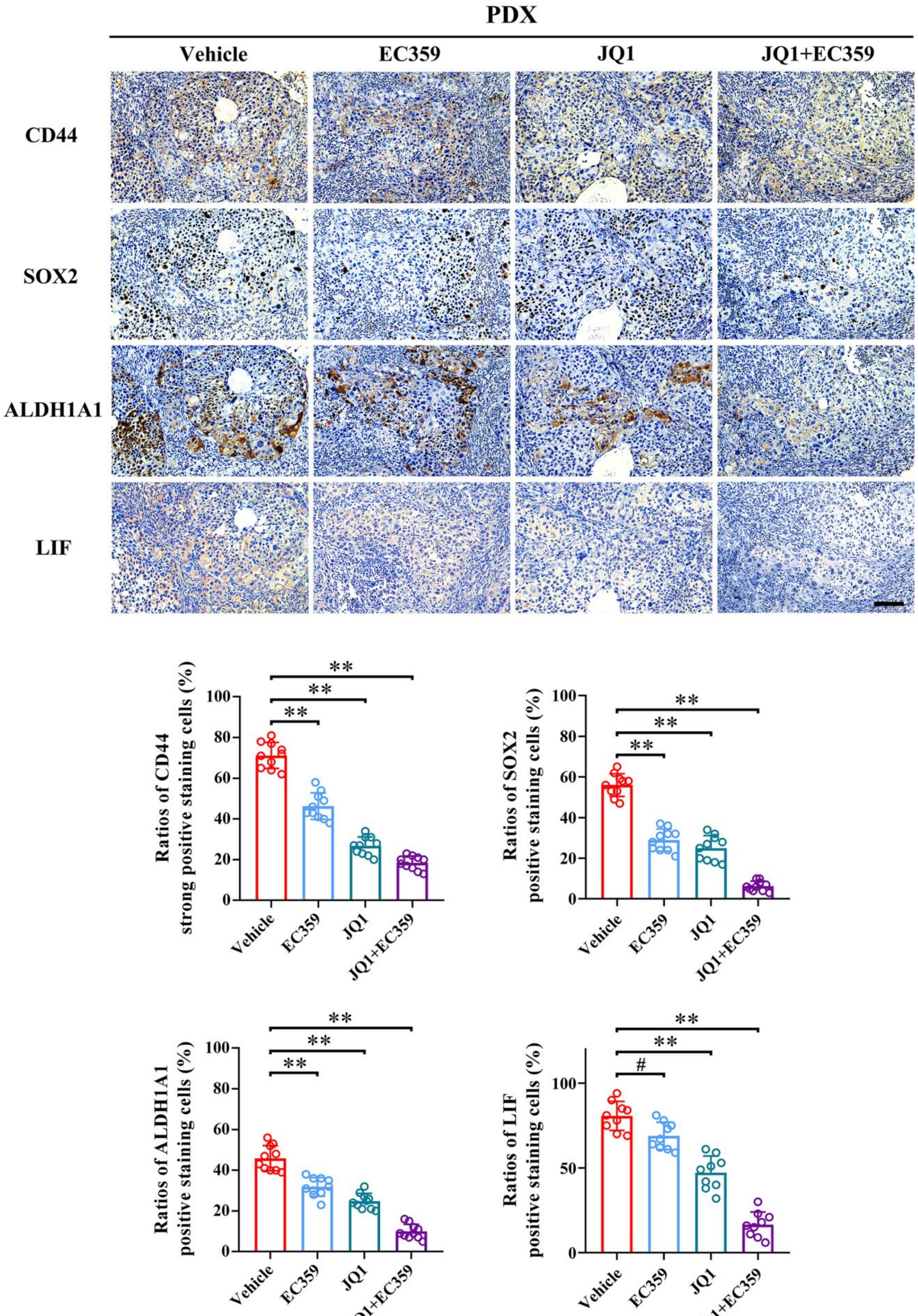

Supplementary Figure 28. (Related to Fig. 7H-I) The combination therapy of JQ1 and EC359 causes

remarkable repression of tumor growth and stemness in HNSCC PDX model.

Representative IHC staining images and corresponding quantification data of CD44, SOX2, ALDH1A1 and LIF in PDX samples treated with vehicle, EC359 or JQ1 alone, or in combination were shown. Scale bar: 100 μm. Data were presented as Mean ± SD. Student's *t* test. $^{\#}P \geqslant 0.05$, $^{**}P < 0.01$.
